# Supplementary material for: PML restrains p53 activity and cellular senescence in clear cell renal cell carcinoma
Source: EMBO Mol Med. 2024 May 10;16(6):7. doi: 10.1038/s44321-024-00077-3 (PMC11178789; doi:10.1038/s44321-024-00077-3)
Supplement: Supplementary file 1 — Appendix [file 44321_2024_77_MOESM1_ESM.pdf]

Appendix

PML restrains p53 activity and cellular senescence in clear cell renal cell carcinoma

Table of contents

Appendix Figure S1. PML depleted ccRCC cell lines are not SA-b-gal positive at 96 hours of doxycycline induction……………………………………………..……………………………2

Appendix Figure S2. PML silencing does not induce DNA damage in ccRCC cells…………..……3

Appendix Supplementary Methods. Pipeline used for calculation of PML distribution if or outside PML-NBs in RCC4 and MDA-MB-231 cells………………..………………………………………4

Appendix Figure S1

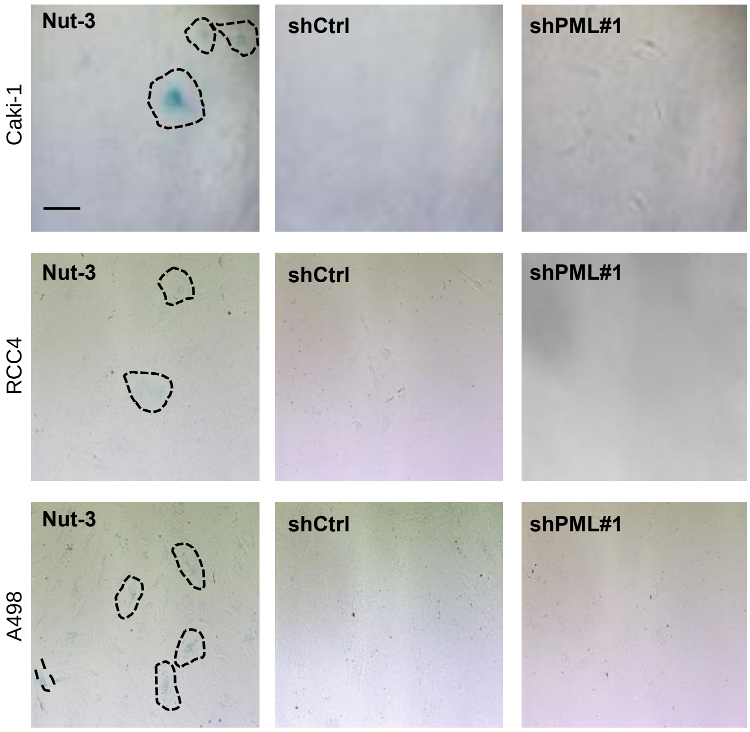

Appendix Figure S1. PML depleted ccRCC cell lines are not SA-b-gal positive at 96 hours of doxycycline induction. Representative phase contrast images of the indicated cell lines expressing shPML#1 or shCtrl or treated with 10 nM Nutlin-3 (Nut-3) stained for SA-β-gal. Scale bar 20 µm. Shown are the results of one out of three experiment.

Appendix Figure S2.

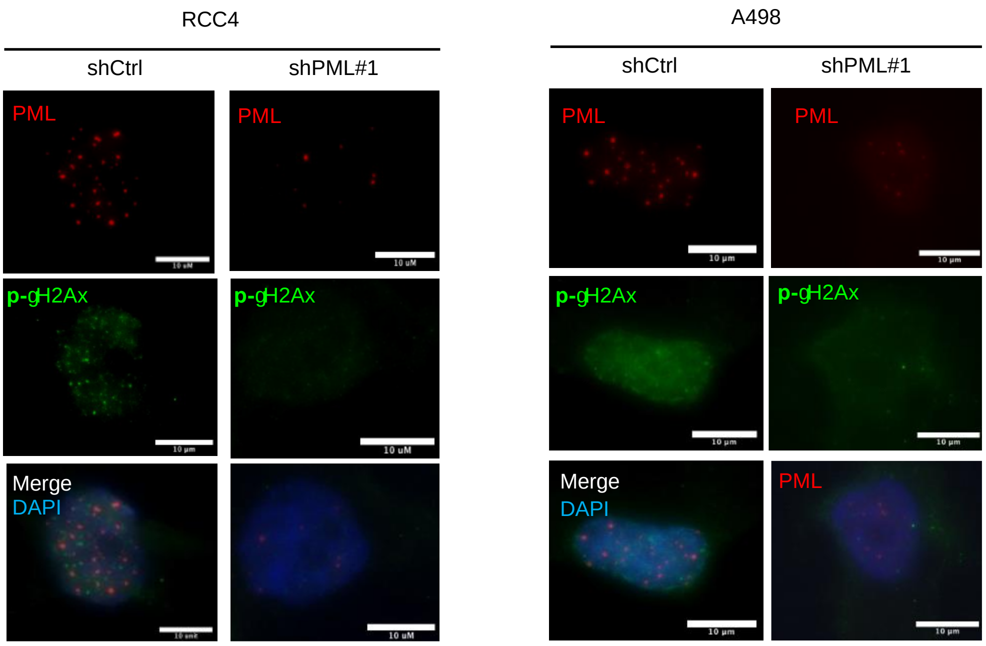

Appendix Figure S2. PML silencing does not induce DNA damage in ccRCC cells. Representative p-γ-H2AX (green), PML (red) immunofluorescence images of RCC4 and A498 cell lines expressing shPML#1 or shCtrl. Nuclei were counterstained with DAPI (blue). Scalebar 10 µm. Shown are the results of one out of three experiment.

Appendix Supplementary Methods. Pipeline used for calculation of PML distribution if or outside PML-NBs in RCC4 and MDA-MB-231 cells.

```
clear; close all;
clc

csvSuffix ='20240229_PML_';
ClusterRings = readtable([csvSuffix,'ClusterRings.csv']); % Load Nuclei datasheet
Clusters = readtable([csvSuffix,'Clusters.csv']); % Load Clusters datasheet

varNames = {'Sample', 'FileName','NuclId', 'ClustId', ...
   'RedIn', 'BlueIn', 'RedOut',  'BlueOut', 'ClusterSize'};
varTypes = {'double', 'string','double'  'double', 'double','double',...
   'double','double','double'};

SummaryTable = table('Size', [height(Clusters),numel(varTypes)], 'VariableTypes',varTypes, 'VariableNames',varNames);

%%
%cycle on Cluster

SummaryTable.FileName = Clusters.FileName_RAW;
SummaryTable.Sample =  1*contains(Clusters.FileName_RAW,'RCC4') + ...
   2*contains(Clusters.FileName_RAW,'MDA-MB-231');
SummaryTable.NuclId = Clusters.Parent_Nuclei;
SummaryTable.ClustId = Clusters.ObjectNumber;
SummaryTable.BlueIn = Clusters.Intensity_MeanIntensity_OrigBlue_bkg_sub;
SummaryTable.RedIn = Clusters.Intensity_MeanIntensity_OrigRed_bkg_sub;
SummaryTable.BlueOut = ClusterRings.Intensity_MeanIntensity_OrigBlue_bkg_sub;
SummaryTable.RedOut = ClusterRings.Intensity_MeanIntensity_OrigRed_bkg_sub;
SummaryTable.ClusterSize = Clusters.AreaShape_Area;

%%
%cluster-bycluster (log2 outside over inside) [R,G,B order];
log2_I_O = log2(SummaryTable{:,5:6}./SummaryTable{:,7:8});

%%
VarNamesToPlot ={'log_2(PML_{IN}/PML_{OUT})', 'log_2(DAPI_{IN}/DAPI_{OUT})'};

h = figure;
h.Position = [300,300,792,308];
for i = 1:2
   Dum = iosr.statistics.tab2box(SummaryTable.Sample, log2_I_O(:,i));
   haxes = subplot(1,3,i);
   iosr.statistics.boxPlot(Dum, 'showScatter', true, ...
      'outlierSize', 5,'scatterMarker','o','scatterSize', 5,...
      'scatterLayer', 'bottom', 'showViolin', true,...
      'xSpacing', 'x');

   Samples ={'RCC4', 'MDA-MB-231'};
```

```
    xticks([1 2]);
    xticklabels(Samples);
    box on;
    title(VarNamesToPlot(i));
    ylabel(VarNamesToPlot(i));

    hold on;
    plot([0.5 3.5], [0 0], '--k', 'LineWidth', 1.5);
    hold off;

    doKStest(haxes, log2_I_O(SummaryTable.Sample==1&isfinite(log2_I_O(:,i)),i), ...
        log2_I_O(SummaryTable.Sample==2&isfinite(log2_I_O(:,i)),i), 1)

end

Dum = iosr.statistics.tab2box(SummaryTable.Sample, SummaryTable.ClusterSize);

  haxes = subplot(1,3,3);
    iosr.statistics.boxPlot(Dum, 'showScatter', true, ...
        'outlierSize', 5,'scatterMarker','o','scatterSize', 5,...
        'scatterLayer', 'bottom', 'showViolin', true,...
        'xSpacing', 'x');

    Samples ={'RCC4', 'MDA-MB-231'};

    xticks([1 2]);
    xticklabels(Samples);
    box on;
    title('Cluster Area [px^2]');
    ylabel('Cluster Area [px^2]');
 doKStest(haxes, SummaryTable.ClusterSize(SummaryTable.Sample==1),...
     SummaryTable.ClusterSize(SummaryTable.Sample==2), 1)

saveas(h, 'ViolinsIn-Out.fig');

%% Number of clusters per cell

Nuclei = readtable([csvSuffix,'Nuclei.csv']); % Load Nuclei datasheet

varNames = {'Sample', 'FileName','NuclId', ...
    'NuclArea', 'MeanBlue', 'MeanRed', 'nClusters'};
varTypes = {'double', 'string','double'  'double',...
    'double','double','double'};

NucleiTable = table('Size', [height(Nuclei),numel(varTypes)], 'VariableTypes',varTypes,
'VariableNames',varNames);

NucleiTable.NuclArea = Nuclei.AreaShape_Area;
NucleiTable.MeanBlue = Nuclei.Intensity_MeanIntensity_OrigBlue_bkg_sub;
NucleiTable.MeanRed = Nuclei.Intensity_MeanIntensity_OrigRed_bkg_sub;
NucleiTable.Sample =  1*contains(Nuclei.FileName_RAW,'RCC4') + ...
```

```
    2*contains(Nuclei.FileName_RAW,'MDA-MB-231');
NucleiTable.nClusters = Nuclei.Children_Clusters_Count;
NucleiTable.FileName = Nuclei.FileName_RAW;
NucleiTable.NuclId = Nuclei.ObjectNumber;

%%

h = figure;
h.Position = [300,300,792,308];

Dum = iosr.statistics.tab2box(NucleiTable.Sample, NucleiTable.NuclArea);

haxes=subplot(1,4,1);
iosr.statistics.boxPlot(Dum, 'showScatter', true, ...
    'outlierSize', 5,'scatterMarker','o','scatterSize', 5,...
    'scatterLayer', 'bottom', 'showViolin', true,...
    'xSpacing', 'x');

Samples ={'RCC4', 'MDA-MB-231'};

xticks([1 2]);
xticklabels(Samples);
box on;
title('Nuclei Area [px^2]');
ylabel('Nuclei Area [px^2]');

 doKStest(haxes, NucleiTable.NuclArea(NucleiTable.Sample==1),...
     NucleiTable.NuclArea(NucleiTable.Sample==2), 1)

Dum = iosr.statistics.tab2box(NucleiTable.Sample, NucleiTable.MeanRed);
haxes=subplot(1,4,2);
iosr.statistics.boxPlot(Dum, 'showScatter', true, ...
    'outlierSize', 5,'scatterMarker','o','scatterSize', 5,...
    'scatterLayer', 'bottom', 'showViolin', true,...
    'xSpacing', 'x');

Samples ={'RCC4', 'MDA-MB-231'};

xticks([1 2]);
xticklabels(Samples);
box on;
title('PML Nuclear Intensity [AU]');
ylabel('PML Nuclear Intensity [AU]');


 doKStest(haxes, NucleiTable.MeanRed(NucleiTable.Sample==1),...
     NucleiTable.MeanRed(NucleiTable.Sample==2), 1)

Dum = iosr.statistics.tab2box(NucleiTable.Sample, NucleiTable.nClusters);
haxes=subplot(1,4,3);
iosr.statistics.boxPlot(Dum, 'showScatter', true, ...
```

```
    'outlierSize', 5,'scatterMarker','o','scatterSize', 5,...
    'scatterLayer', 'bottom', 'showViolin', true,...
    'xSpacing', 'x');

Samples ={'RCC4', 'MDA-MB-231'};

xticks([1 2]);
xticklabels(Samples);
box on;
title('Number of Clusters per Nucleus');
ylabel('n. Cluster/Nuclei');

doKStest(haxes, NucleiTable.nClusters(NucleiTable.Sample==1),...
      NucleiTable.nClusters(NucleiTable.Sample==2), 1)

Dum = iosr.statistics.tab2box(NucleiTable.Sample, NucleiTable.nClusters./NucleiTable.NuclArea);
haxes=subplot(1,4,4);
iosr.statistics.boxPlot(Dum, 'showScatter', true, ...
    'outlierSize', 5,'scatterMarker','o','scatterSize', 5,...
    'scatterLayer', 'bottom', 'showViolin', true,...
    'xSpacing', 'x');

Samples ={'RCC4', 'MDA-MB-231'};

xticks([1 2]);
xticklabels(Samples);
box on;
title('Clusters per unit area');
ylabel('Clusters/px^2');

doKStest(haxes,
NucleiTable.nClusters(NucleiTable.Sample==1)./NucleiTable.NuclArea(NucleiTable.Sample==1),.
..

NucleiTable.nClusters(NucleiTable.Sample==2)./NucleiTable.NuclArea(NucleiTable.Sample==2),
1)

function doKStest(handl, first, second, bonferroniCorrection)

[~,p] = kstest2(first, second);
test = p*bonferroniCorrection;

if test > 0.05
    p_string = 'n.s.';
else
    p_string =  ['p = ', num2str(test,1)];
end

text(handl,1.5, 1.1.*max([first;second]),...
    p_string,'HorizontalAlignment','center', 'FontSize',8.5);
end
```
