# Supplementary material for: PML restrains p53 activity and cellular senescence in clear cell renal cell carcinoma
Source: EMBO Mol Med. 2024 May 10;16(6):7. doi: 10.1038/s44321-024-00077-3 (PMC11178789; doi:10.1038/s44321-024-00077-3)
Supplement: Supplementary file 3 — Source data Fig. 1 [file 44321_2024_77_MOESM3_ESM.zip › EMM-2024-19519_SourceDataFor_Figure1/Figure1E_ImageData/ReadMe.docx]

To allow the visualization of nucleoplasmic soluble PML, Brightness/contrast was applied equally to RCC4 and MDA-MB-231 images with the following settings, by using Imgej:

- Minimum displayed value: 229
- Maximum displayed value: 14847
