# Supplementary figures and images for: PML restrains p53 activity and cellular senescence in clear cell renal cell carcinoma
Source: EMBO Mol Med. 2024 May 10;16(6):7. doi: 10.1038/s44321-024-00077-3 (PMC11178789; doi:10.1038/s44321-024-00077-3)

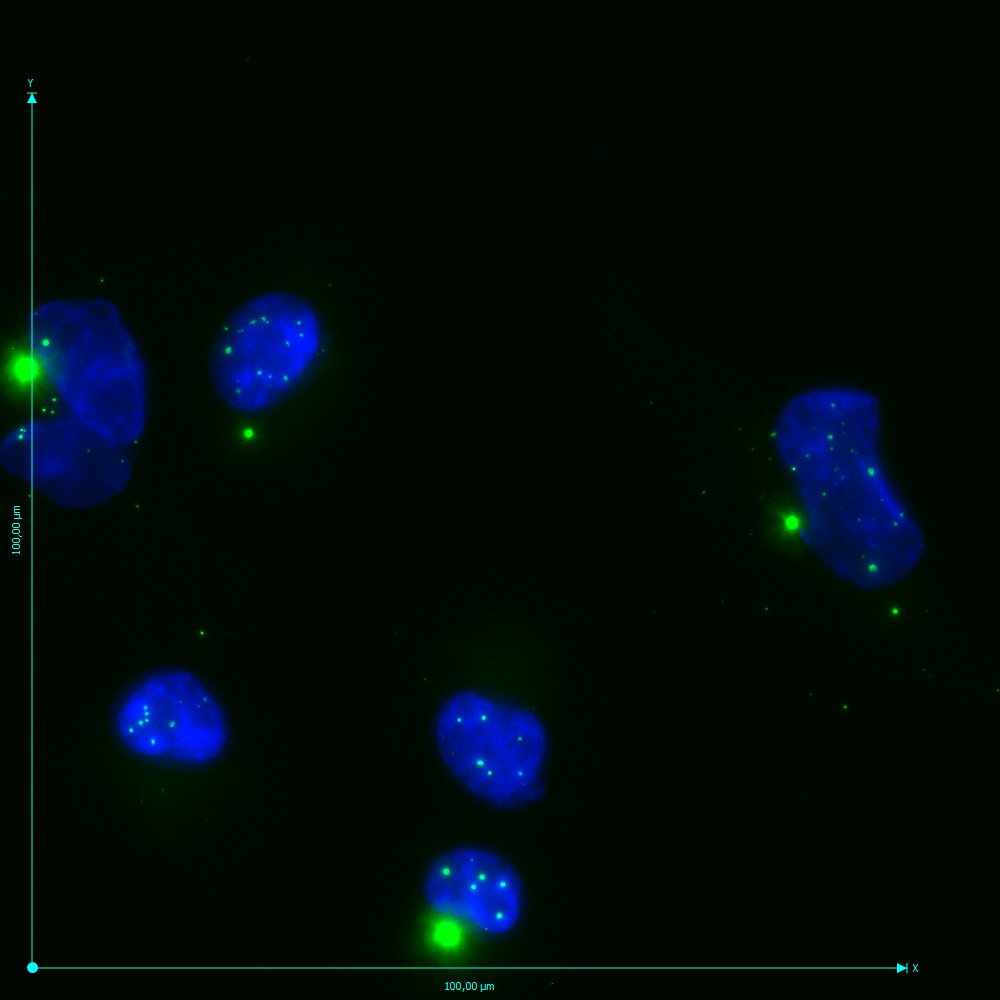

Supplement: Supplementary file 3 — Source data Fig. 1 [file 44321_2024_77_MOESM3_ESM.zip › EMM-2024-19519_SourceDataFor_Figure1/Figure1K_ImageData/MDA-MB-231/MAX_231 ATO Release 6h.jpg]

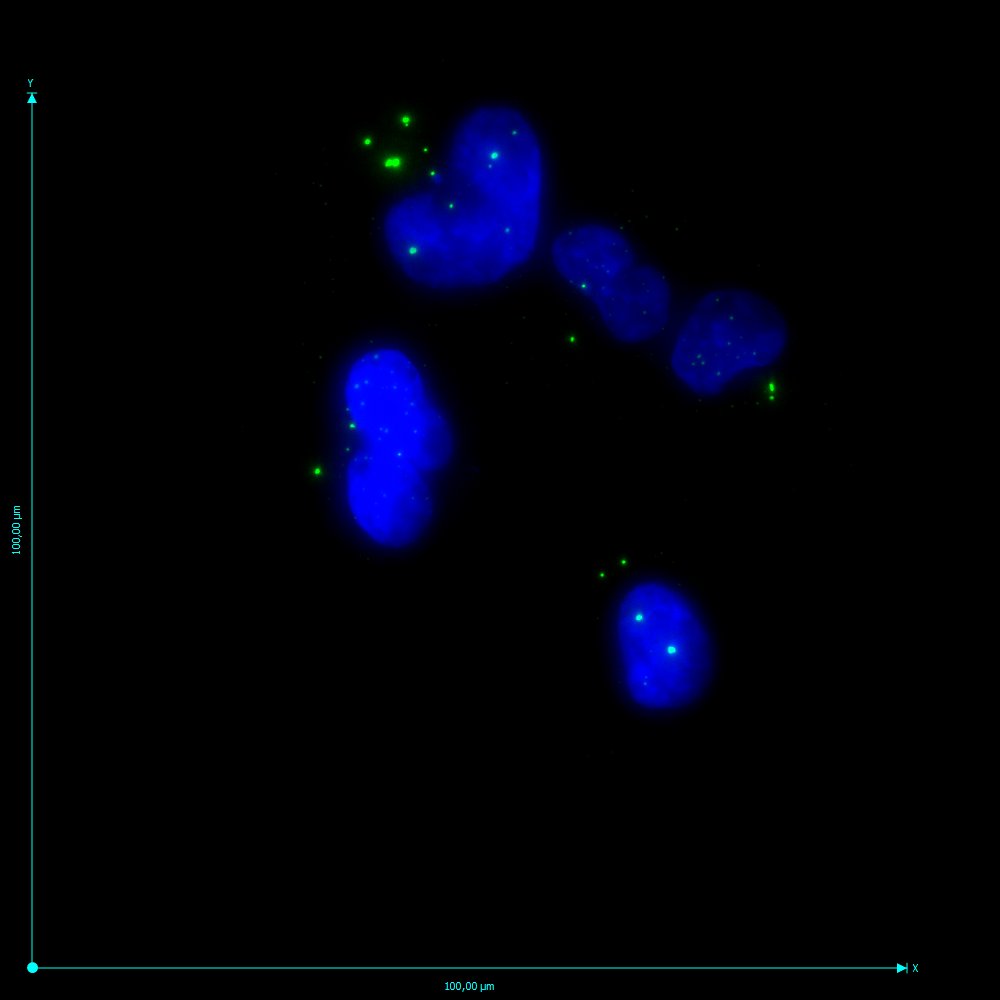

Supplement: Supplementary file 3 — Source data Fig. 1 [file 44321_2024_77_MOESM3_ESM.zip › EMM-2024-19519_SourceDataFor_Figure1/Figure1K_ImageData/MDA-MB-231/MAX_231 Release 3h.jpg]

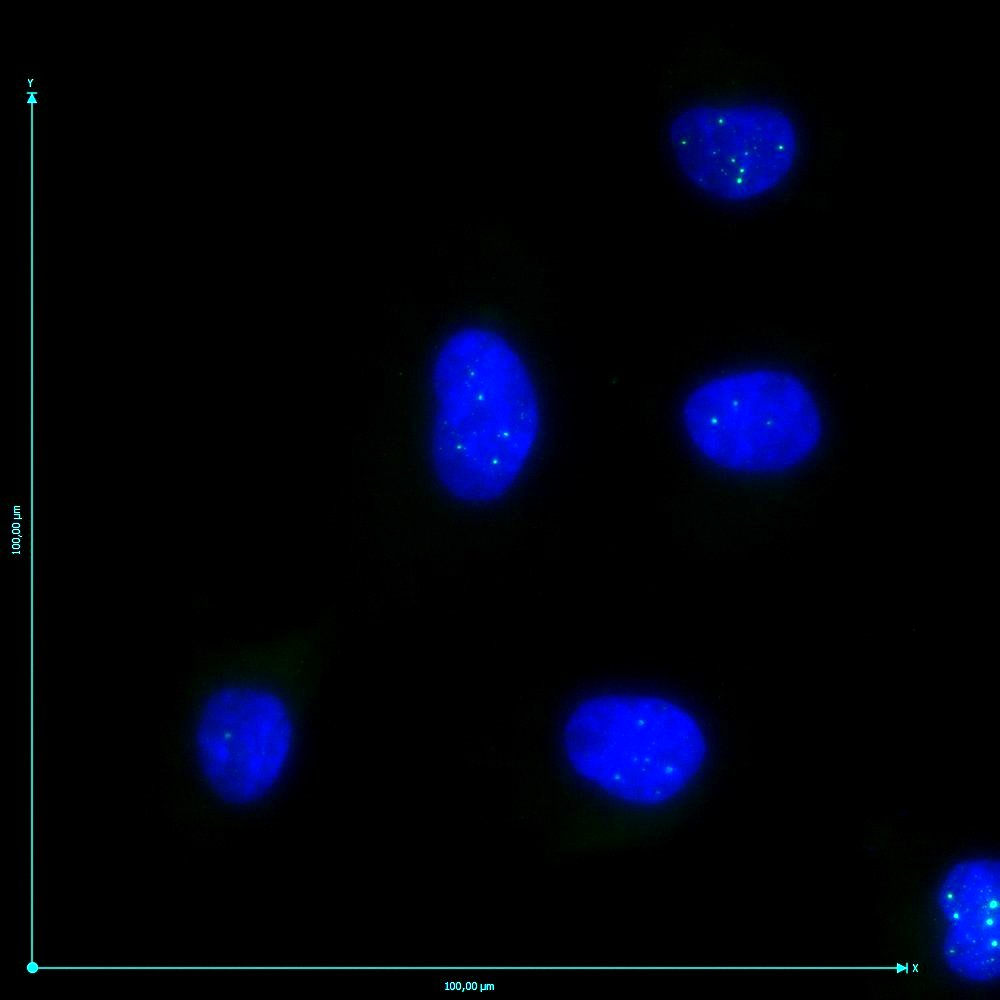

Supplement: Supplementary file 3 — Source data Fig. 1 [file 44321_2024_77_MOESM3_ESM.zip › EMM-2024-19519_SourceDataFor_Figure1/Figure1K_ImageData/MDA-MB-231/MAX_231 Veh.jpg]

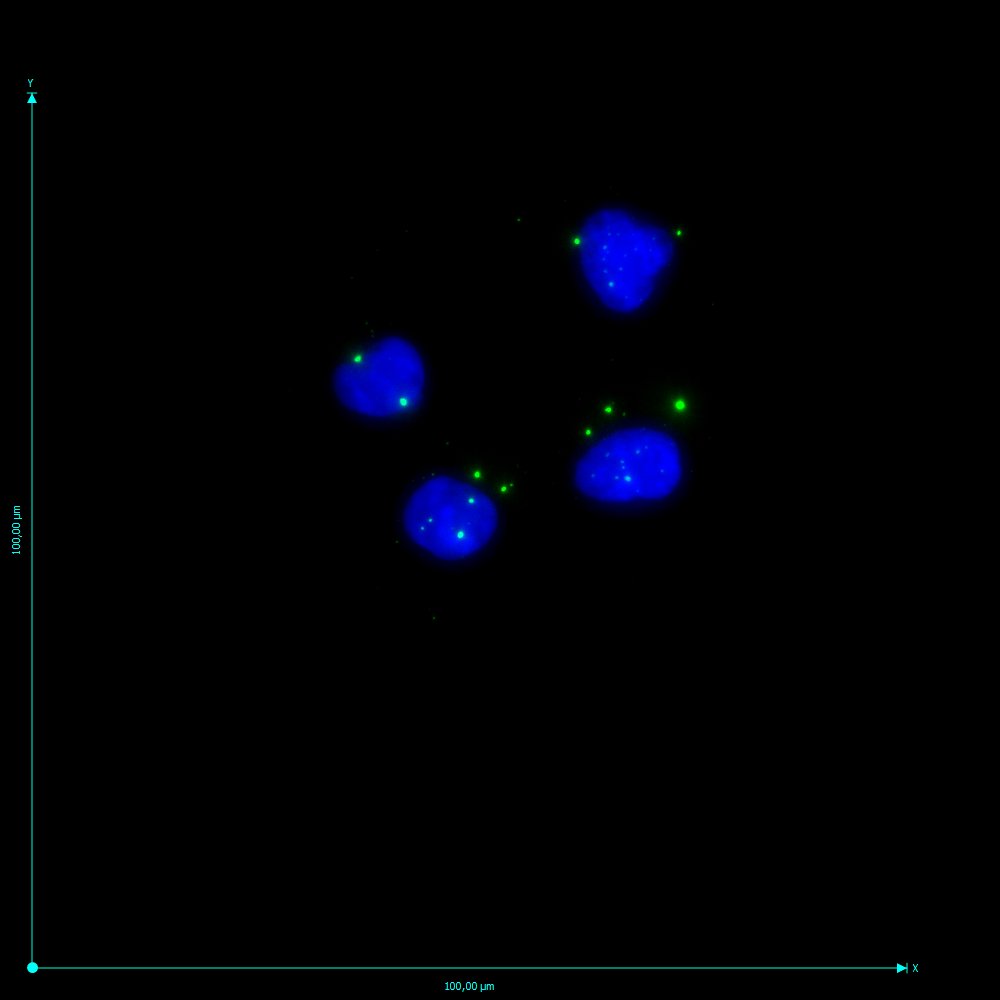

Supplement: Supplementary file 3 — Source data Fig. 1 [file 44321_2024_77_MOESM3_ESM.zip › EMM-2024-19519_SourceDataFor_Figure1/Figure1K_ImageData/MDA-MB-231/MAX_231 ATO 48h.jpg]

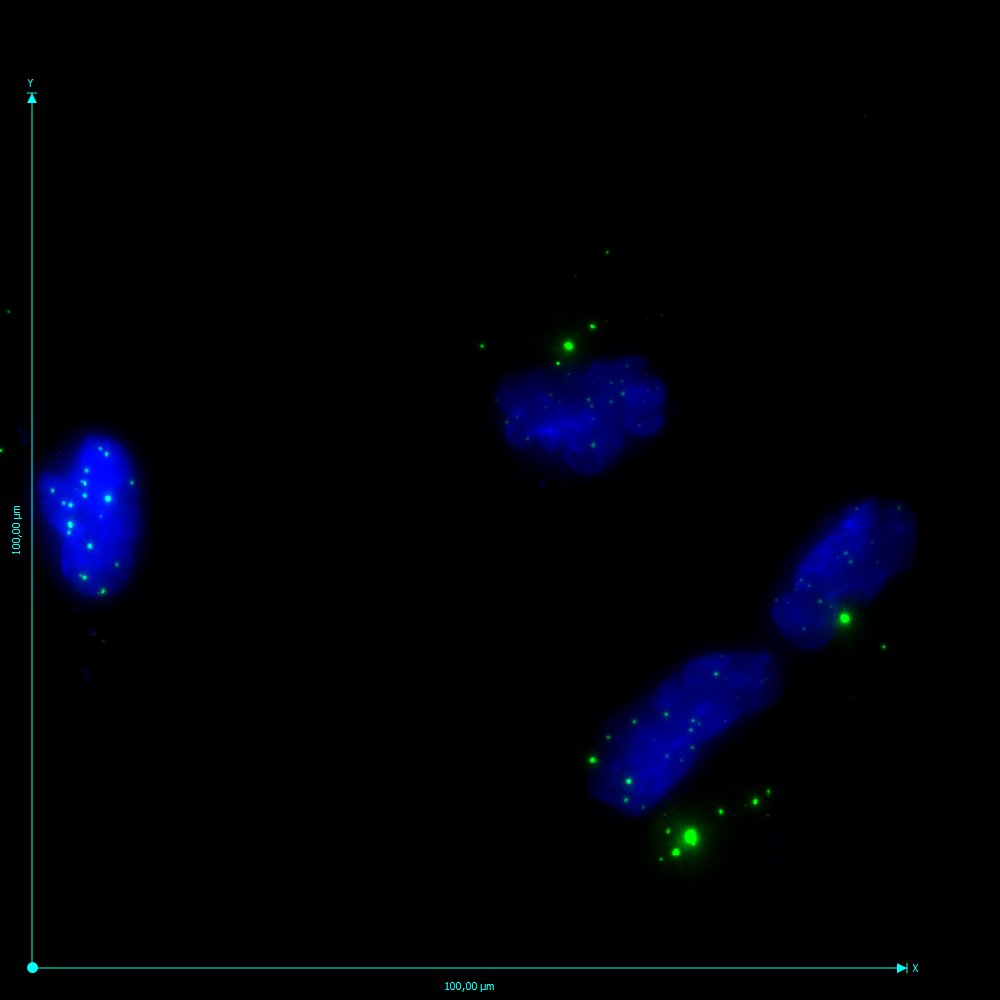

Supplement: Supplementary file 3 — Source data Fig. 1 [file 44321_2024_77_MOESM3_ESM.zip › EMM-2024-19519_SourceDataFor_Figure1/Figure1K_ImageData/RCC4/MAX_RCC4 Release 3h.jpg]

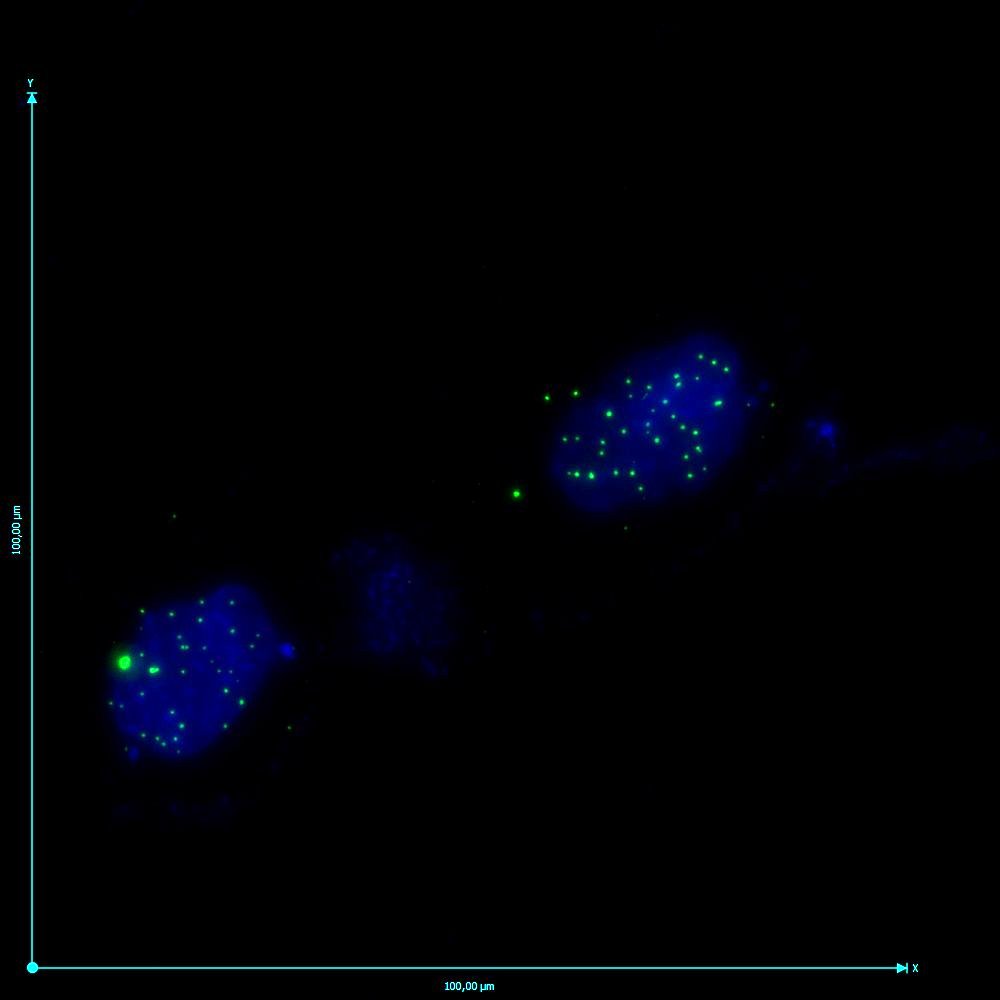

Supplement: Supplementary file 3 — Source data Fig. 1 [file 44321_2024_77_MOESM3_ESM.zip › EMM-2024-19519_SourceDataFor_Figure1/Figure1K_ImageData/RCC4/RCC4 Release 6h .jpg]

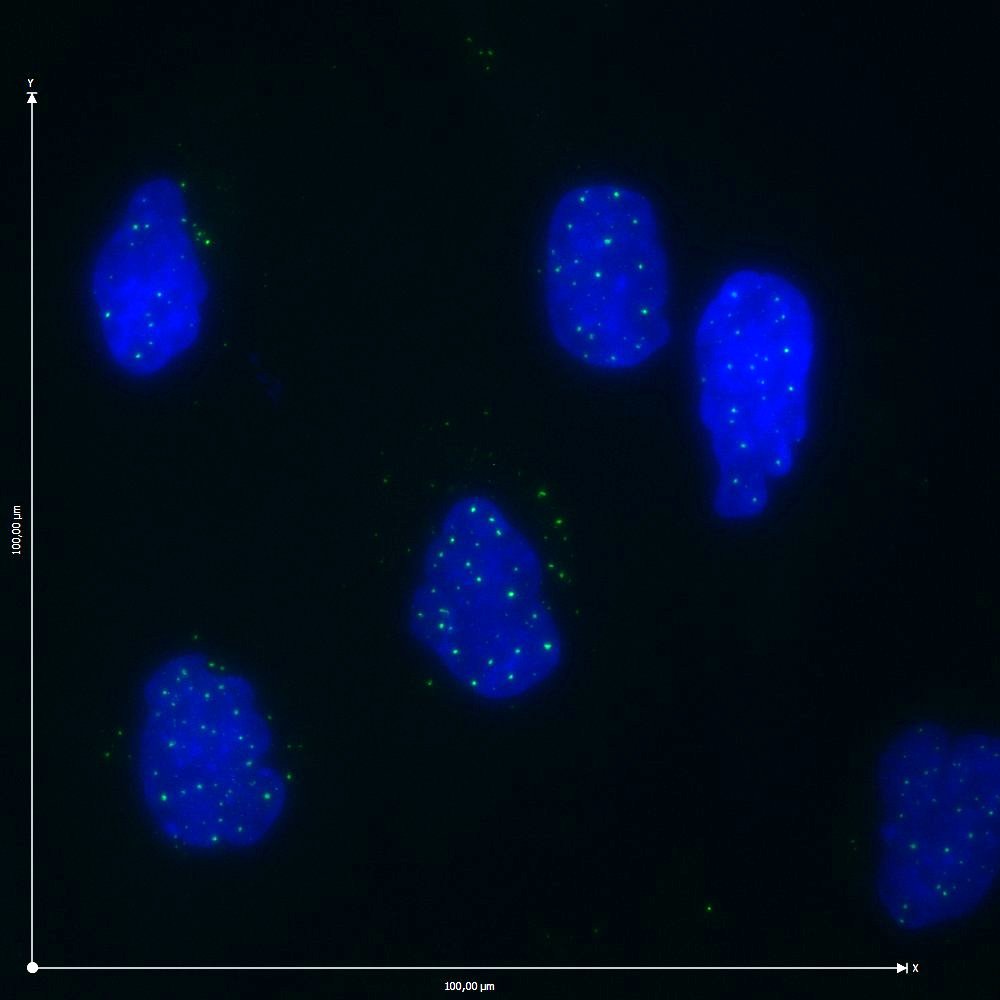

Supplement: Supplementary file 3 — Source data Fig. 1 [file 44321_2024_77_MOESM3_ESM.zip › EMM-2024-19519_SourceDataFor_Figure1/Figure1K_ImageData/RCC4/MAX_RCC4 Veh.jpg]

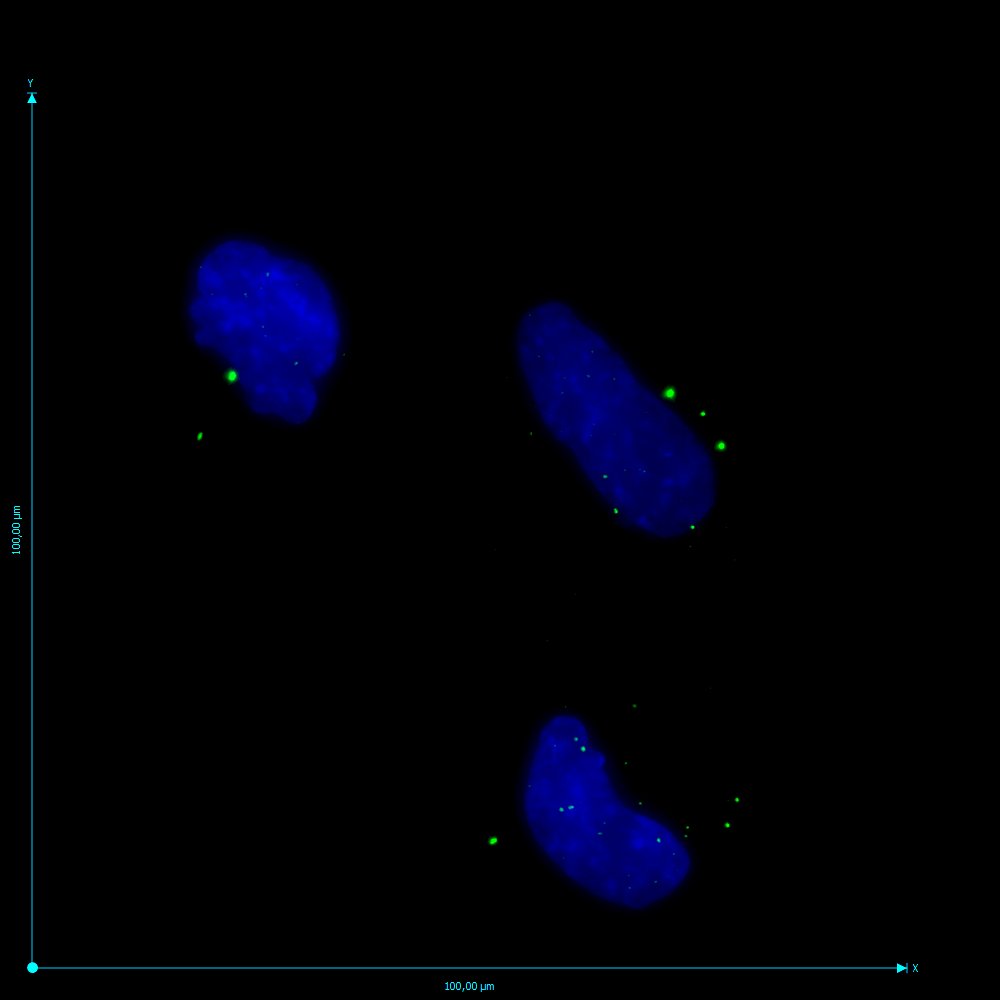

Supplement: Supplementary file 3 — Source data Fig. 1 [file 44321_2024_77_MOESM3_ESM.zip › EMM-2024-19519_SourceDataFor_Figure1/Figure1K_ImageData/RCC4/MAX_RCC4 ATO 48h.jpg]

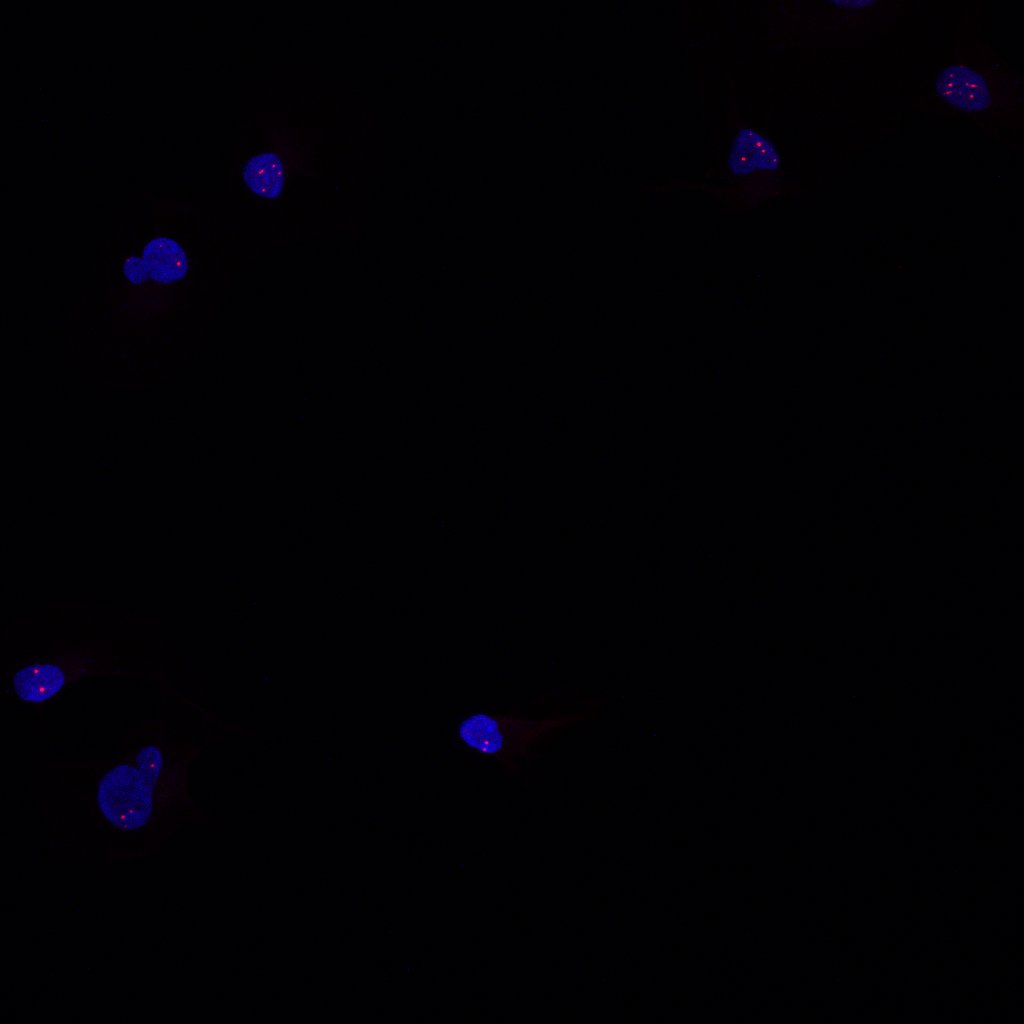

Supplement: Supplementary file 3 — Source data Fig. 1 [file 44321_2024_77_MOESM3_ESM.zip › EMM-2024-19519_SourceDataFor_Figure1/Figure1E_ImageData/MAX_MDA-MB-231.jpg]

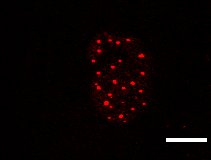

Supplement: Supplementary file 3 — Source data Fig. 1 [file 44321_2024_77_MOESM3_ESM.zip › EMM-2024-19519_SourceDataFor_Figure1/Figure1E_ImageData/ZoomIn_RCC4.jpg]

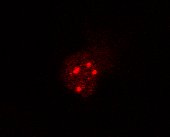

Supplement: Supplementary file 3 — Source data Fig. 1 [file 44321_2024_77_MOESM3_ESM.zip › EMM-2024-19519_SourceDataFor_Figure1/Figure1E_ImageData/ZoomIn_MDA-MB-231_4.jpg]

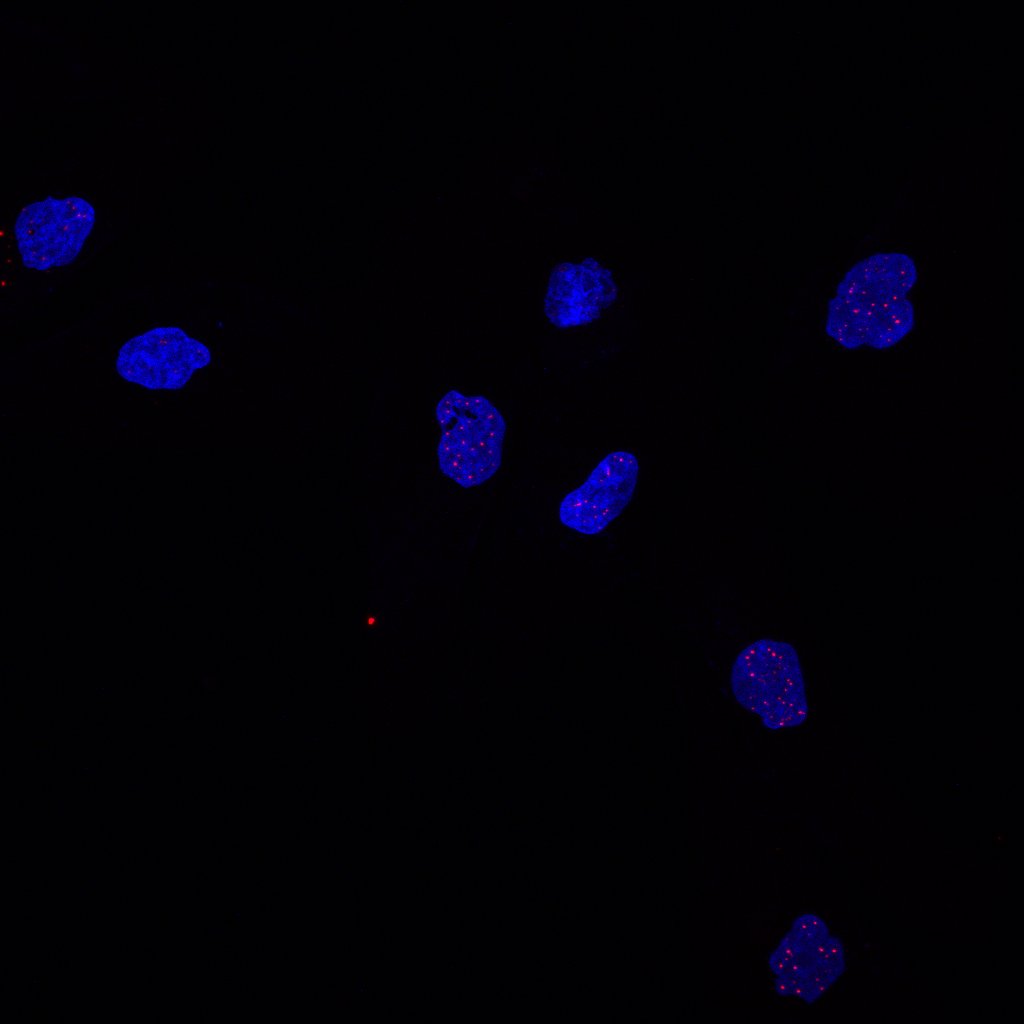

Supplement: Supplementary file 3 — Source data Fig. 1 [file 44321_2024_77_MOESM3_ESM.zip › EMM-2024-19519_SourceDataFor_Figure1/Figure1E_ImageData/MAX_RCC4.jpg]

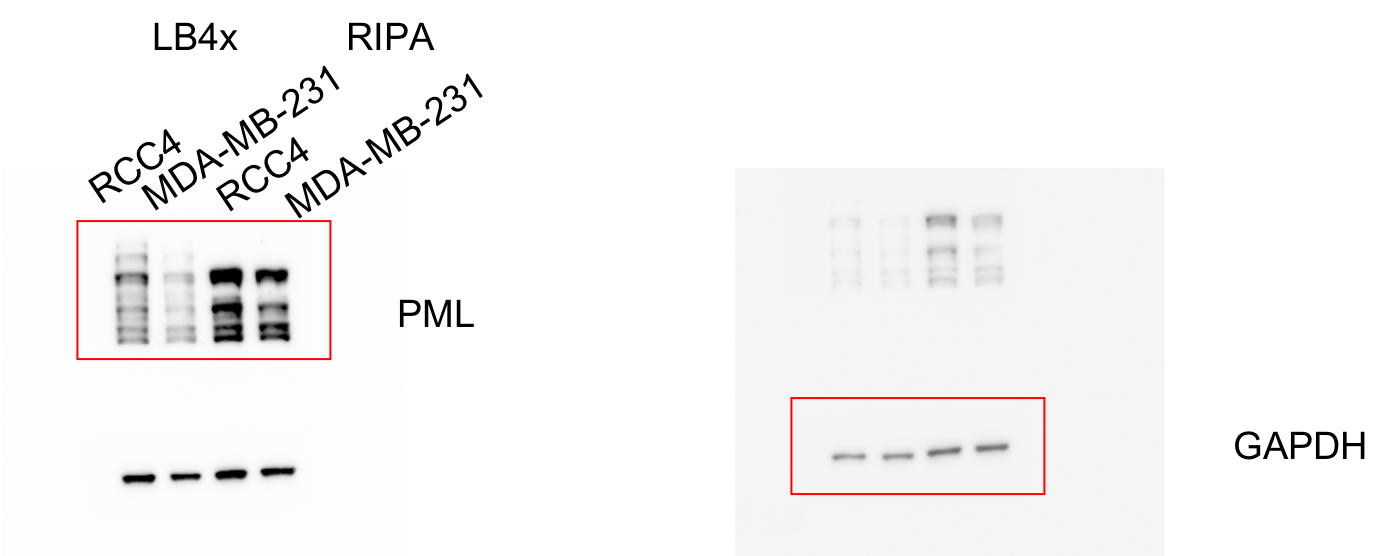

Supplement: Supplementary file 3 — Source data Fig. 1 [file 44321_2024_77_MOESM3_ESM.zip › EMM-2024-19519_SourceDataFor_Figure1/Figure1F.png]

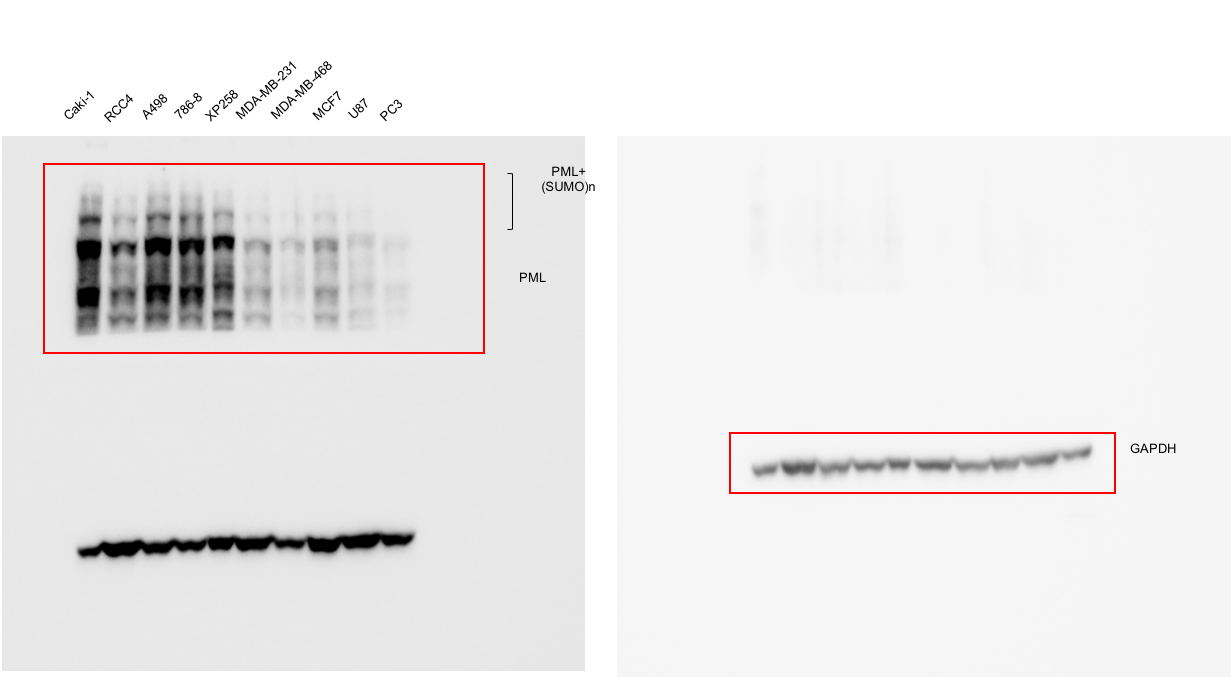

Supplement: Supplementary file 3 — Source data Fig. 1 [file 44321_2024_77_MOESM3_ESM.zip › EMM-2024-19519_SourceDataFor_Figure1/Figure1A.png]

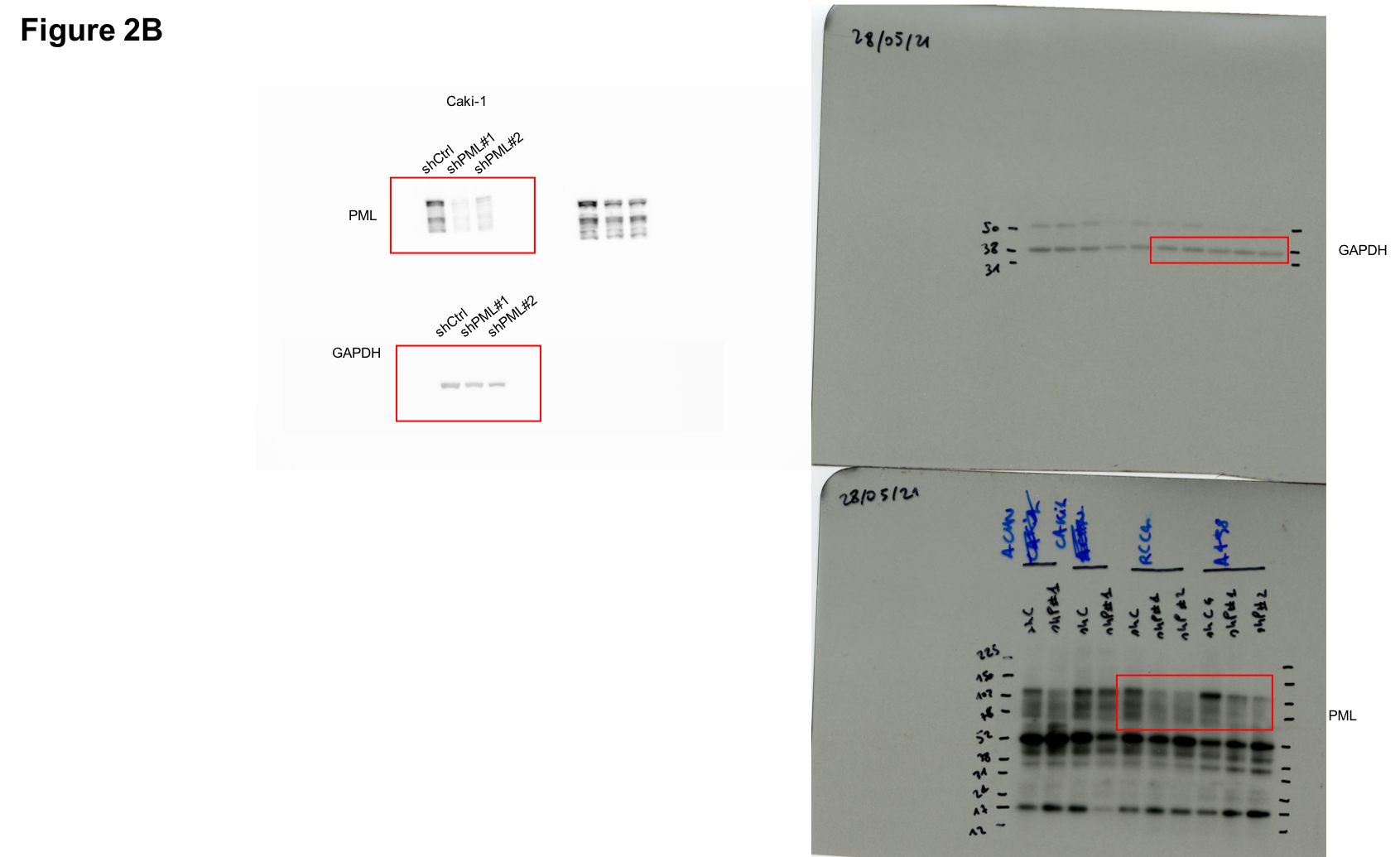

Supplement: Supplementary file 4 — Source data Fig. 2 [file 44321_2024_77_MOESM4_ESM.zip › EMM-2024-19519_SourceDataFor_Figure2/Figure 2B.png]

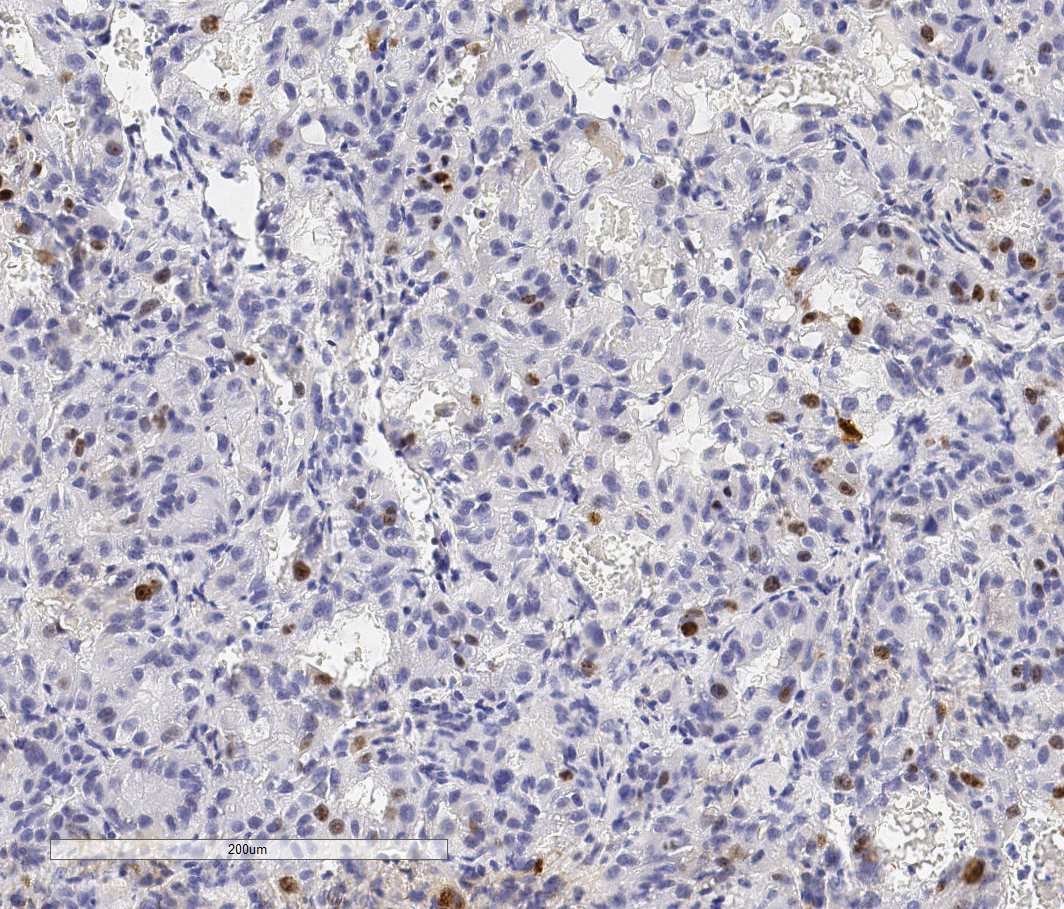

Supplement: Supplementary file 5 — Source data Fig. 3 [file 44321_2024_77_MOESM5_ESM.zip › EMM-2024-19519_SourceDataFor_Figure3/Figure 3F_Image Data/shCtrl.tif]

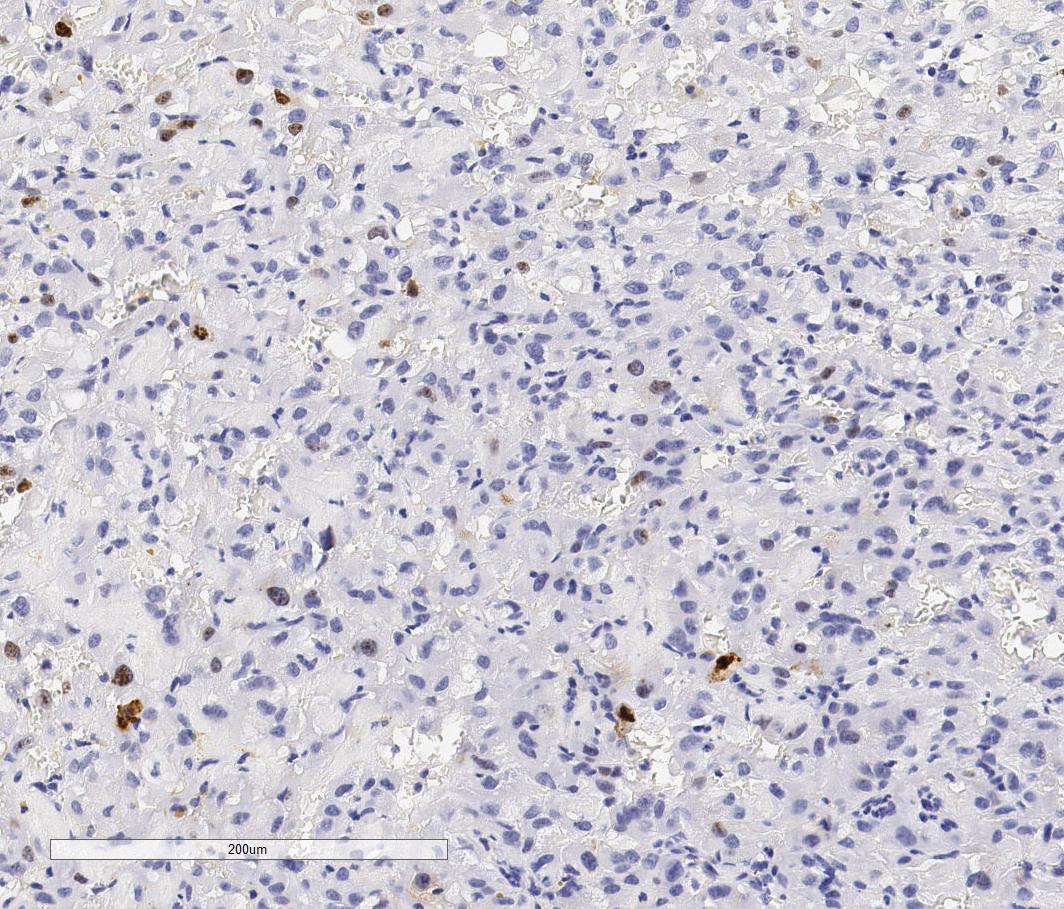

Supplement: Supplementary file 5 — Source data Fig. 3 [file 44321_2024_77_MOESM5_ESM.zip › EMM-2024-19519_SourceDataFor_Figure3/Figure 3F_Image Data/shPML.tif]

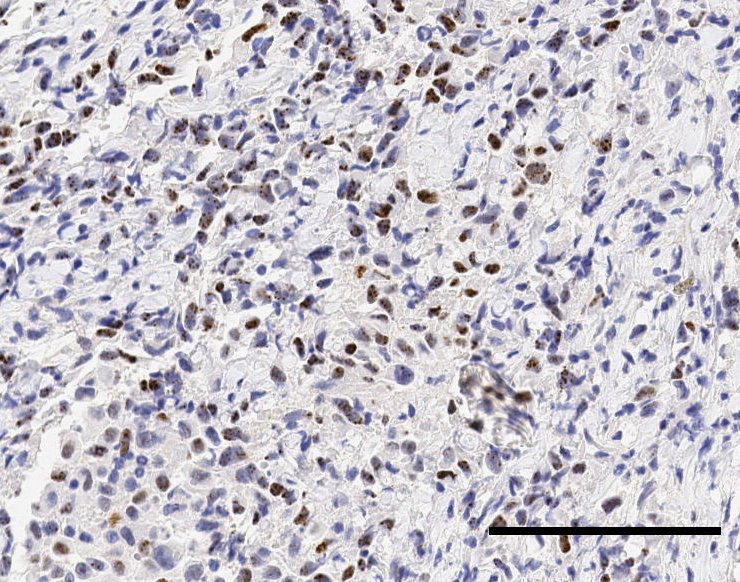

Supplement: Supplementary file 5 — Source data Fig. 3 [file 44321_2024_77_MOESM5_ESM.zip › EMM-2024-19519_SourceDataFor_Figure3/Figure 3C_Image Data/shCtrl.jpg]

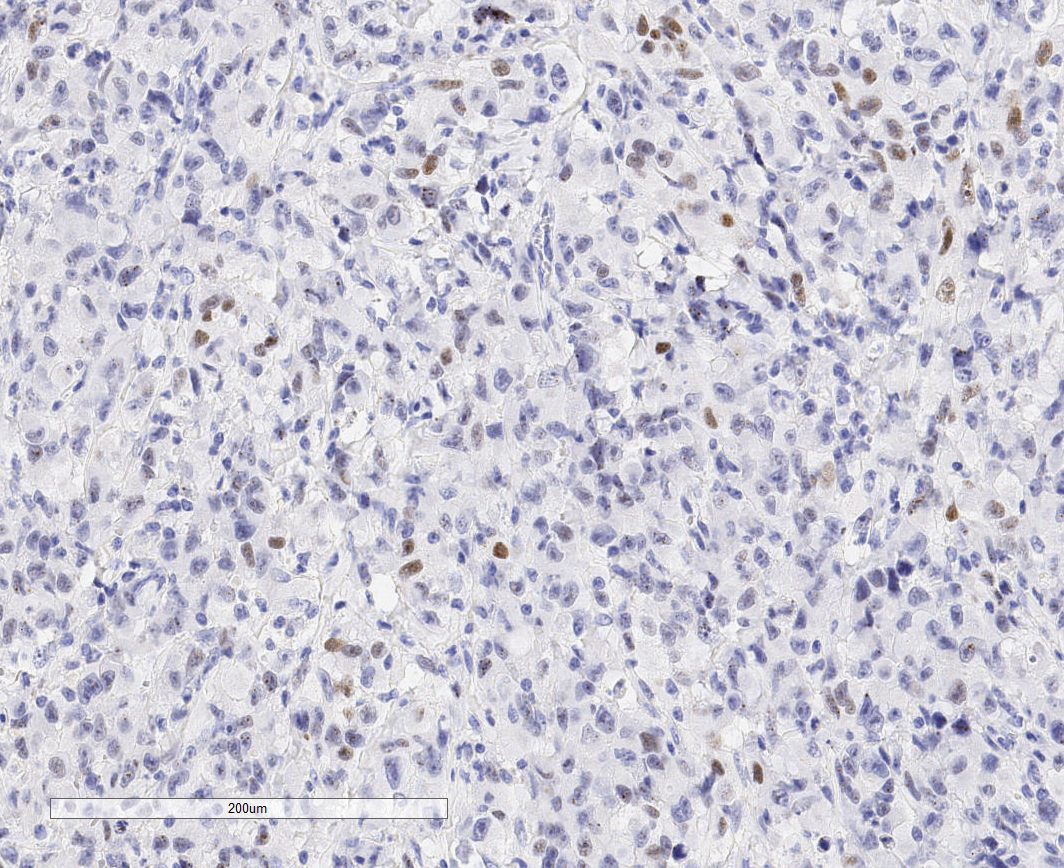

Supplement: Supplementary file 5 — Source data Fig. 3 [file 44321_2024_77_MOESM5_ESM.zip › EMM-2024-19519_SourceDataFor_Figure3/Figure 3C_Image Data/shPML.tif]

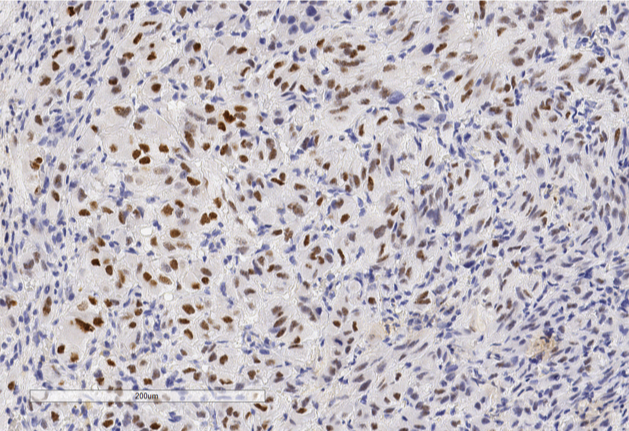

Supplement: Supplementary file 5 — Source data Fig. 3 [file 44321_2024_77_MOESM5_ESM.zip › EMM-2024-19519_SourceDataFor_Figure3/Figure 3D_Image Data/shCtrl.png]

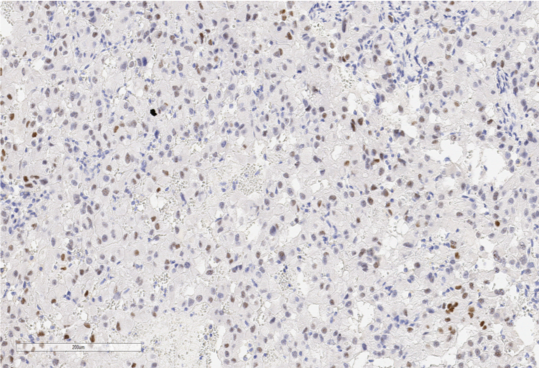

Supplement: Supplementary file 5 — Source data Fig. 3 [file 44321_2024_77_MOESM5_ESM.zip › EMM-2024-19519_SourceDataFor_Figure3/Figure 3D_Image Data/shPML.png]

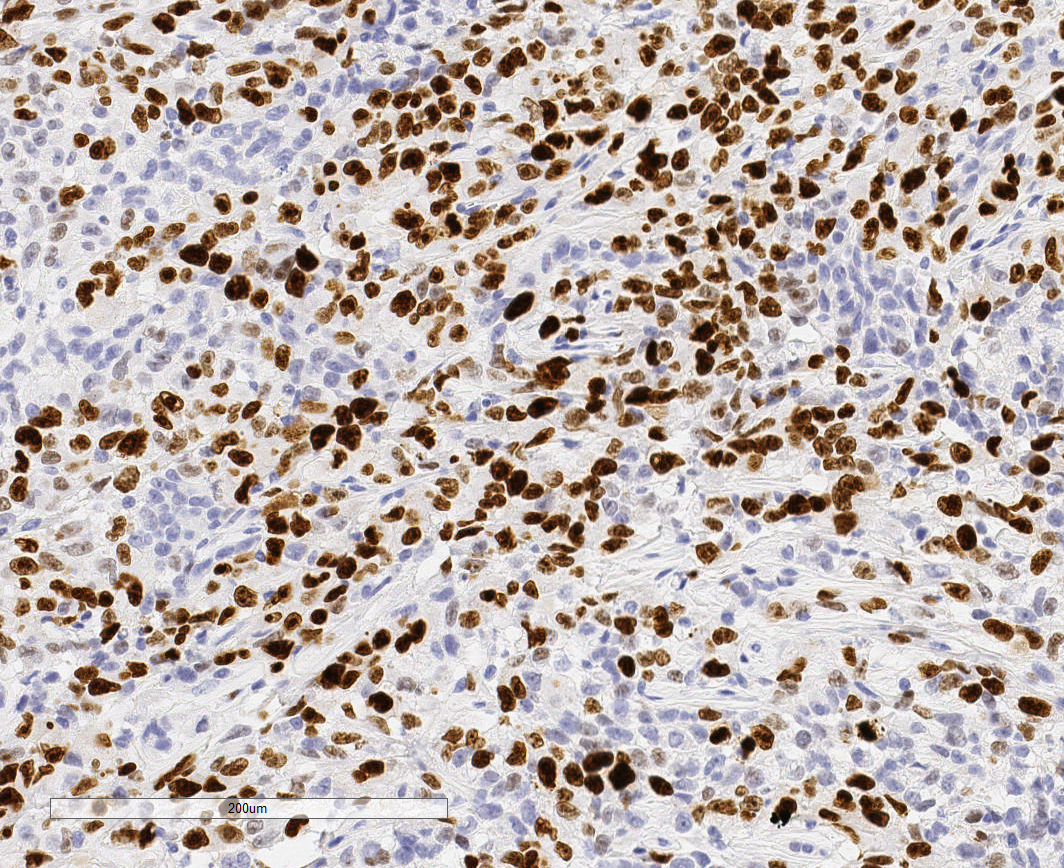

Supplement: Supplementary file 5 — Source data Fig. 3 [file 44321_2024_77_MOESM5_ESM.zip › EMM-2024-19519_SourceDataFor_Figure3/Figure 3E_Image Data/shCtrl.tif]

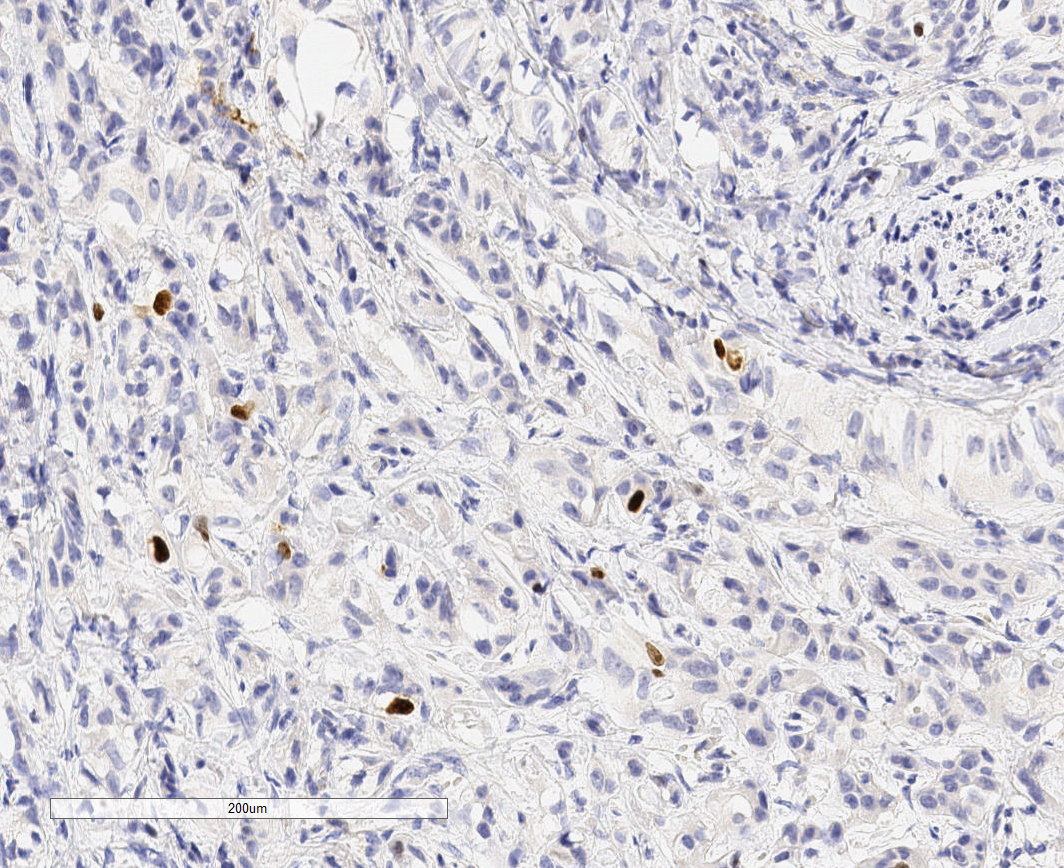

Supplement: Supplementary file 5 — Source data Fig. 3 [file 44321_2024_77_MOESM5_ESM.zip › EMM-2024-19519_SourceDataFor_Figure3/Figure 3E_Image Data/shPML.tif]

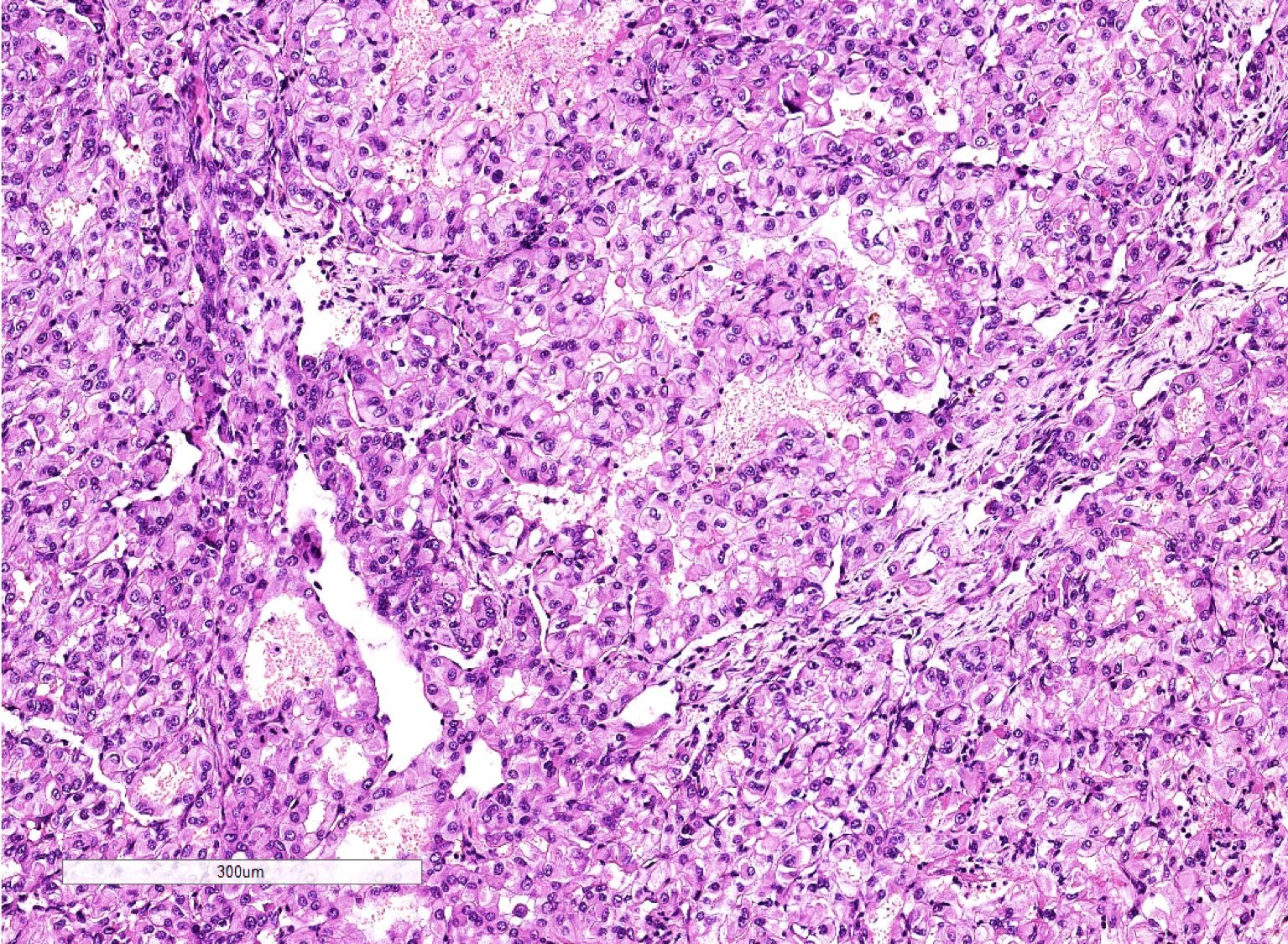

Supplement: Supplementary file 5 — Source data Fig. 3 [file 44321_2024_77_MOESM5_ESM.zip › EMM-2024-19519_SourceDataFor_Figure3/Figure 3G/shPML_ClearCell.png]

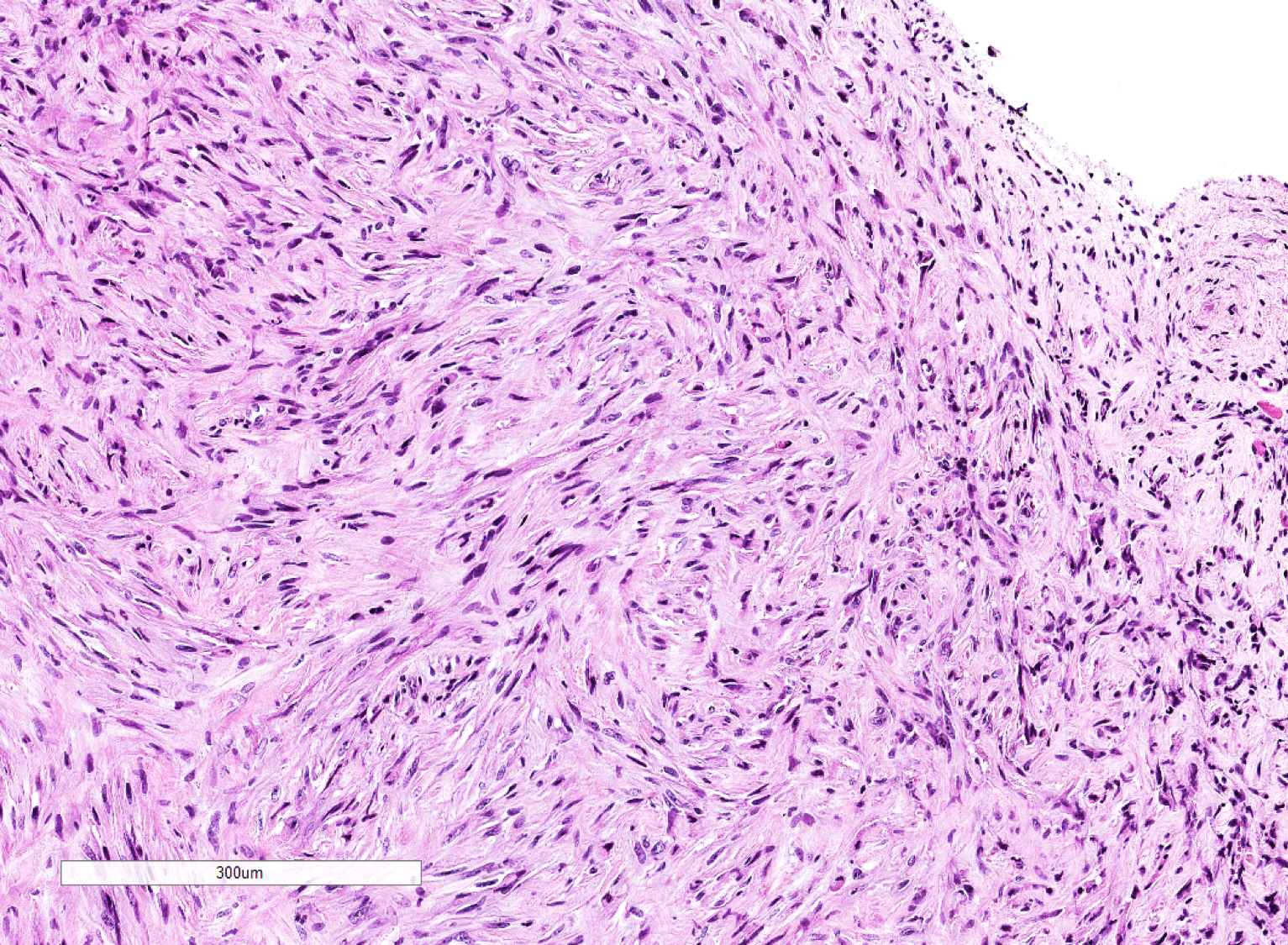

Supplement: Supplementary file 5 — Source data Fig. 3 [file 44321_2024_77_MOESM5_ESM.zip › EMM-2024-19519_SourceDataFor_Figure3/Figure 3G/shPML_Fibrosis.png]

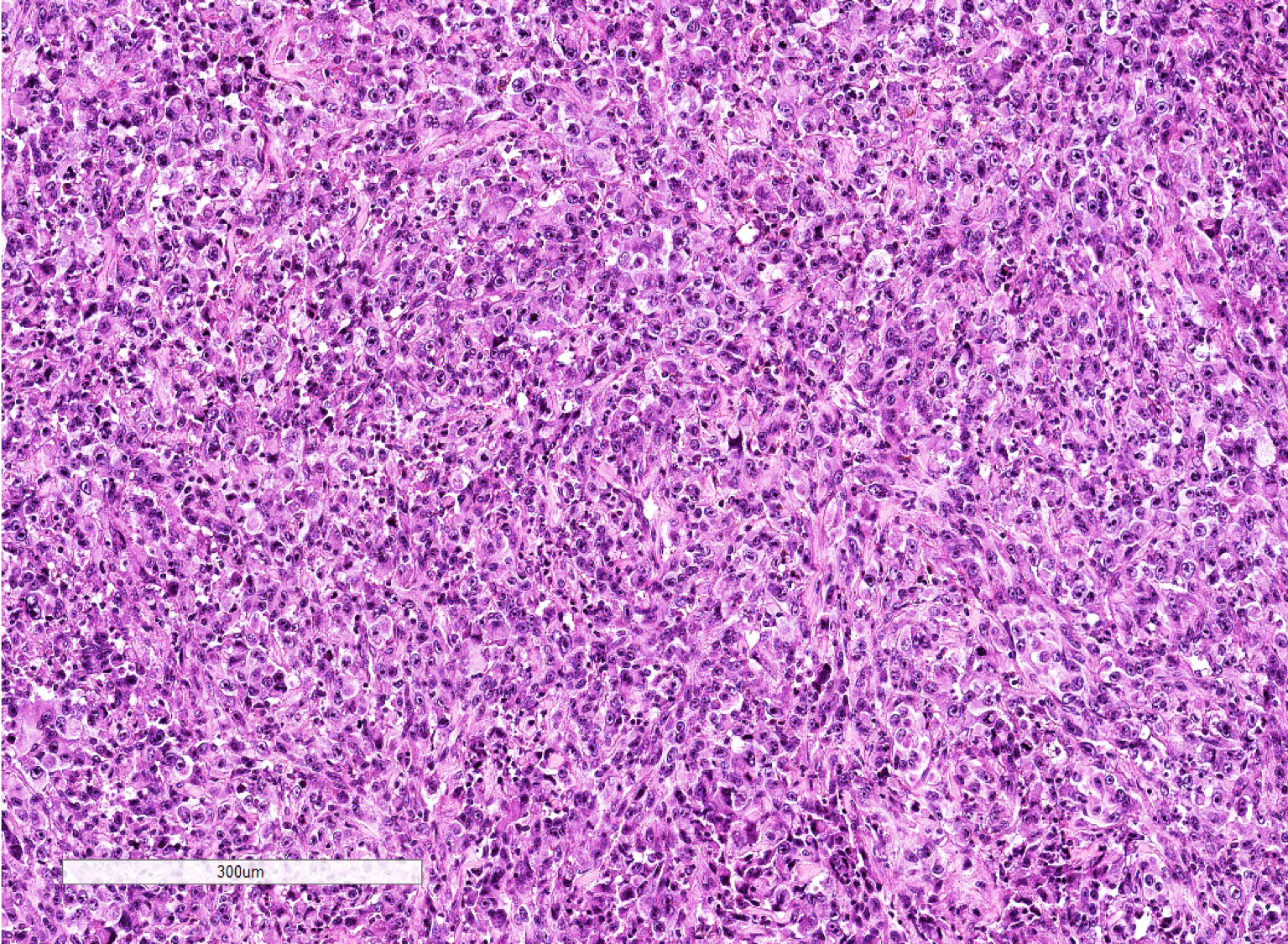

Supplement: Supplementary file 5 — Source data Fig. 3 [file 44321_2024_77_MOESM5_ESM.zip › EMM-2024-19519_SourceDataFor_Figure3/Figure 3G/shCtrl_rhabdoid.png]

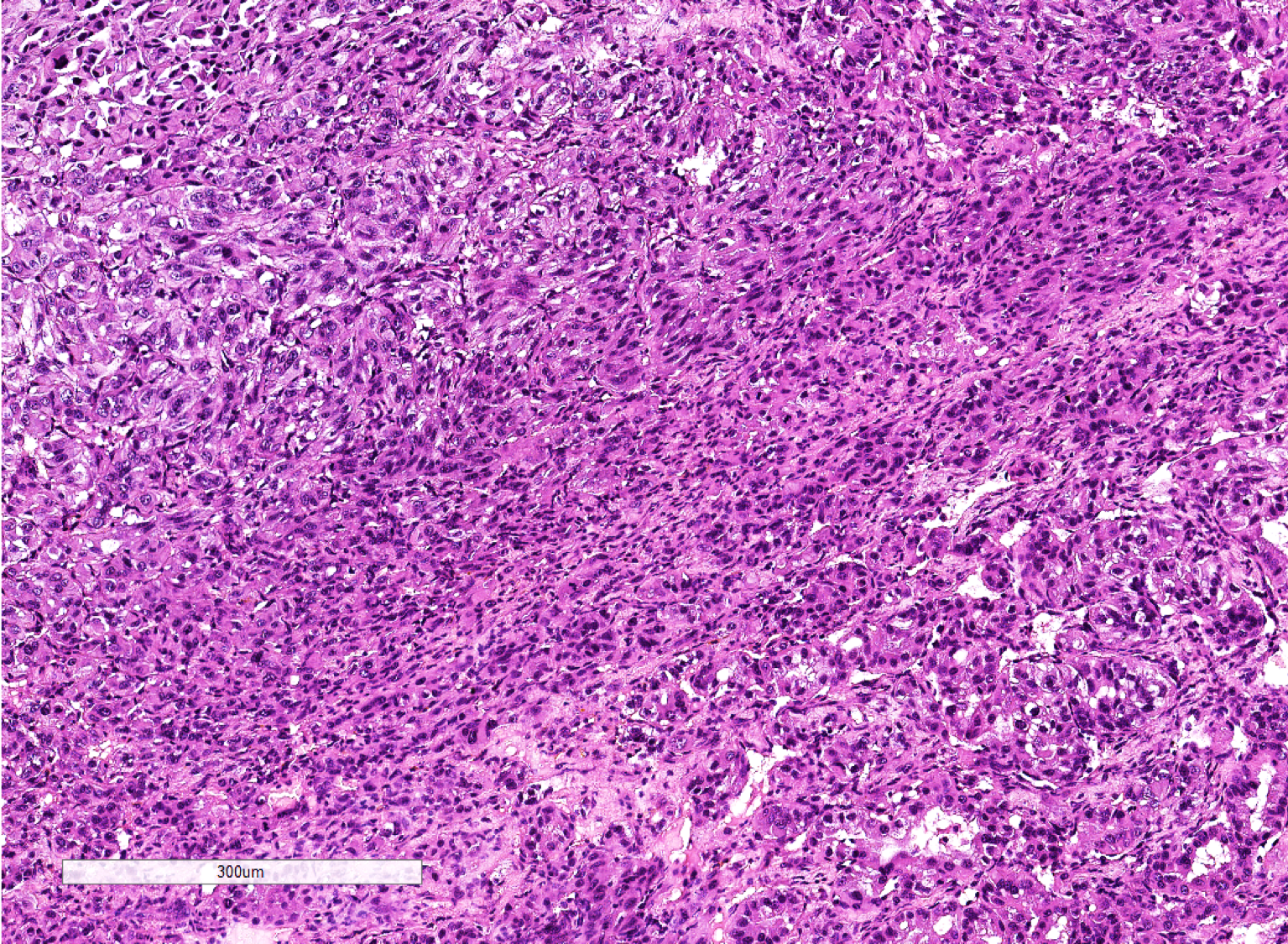

Supplement: Supplementary file 5 — Source data Fig. 3 [file 44321_2024_77_MOESM5_ESM.zip › EMM-2024-19519_SourceDataFor_Figure3/Figure 3G/shCtrl_sarcomatoid.png]

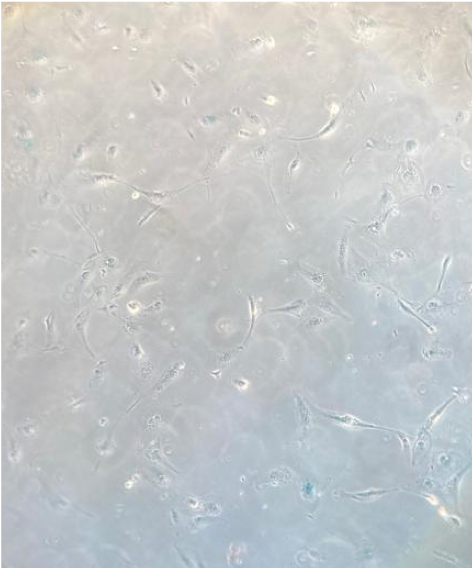

Supplement: Supplementary file 6 — Source data Fig. 5 [file 44321_2024_77_MOESM6_ESM.zip › EMM-2024-19519_SourceDataFor_Figure5/Fugure 5D_Image Data/shCtrl.png]

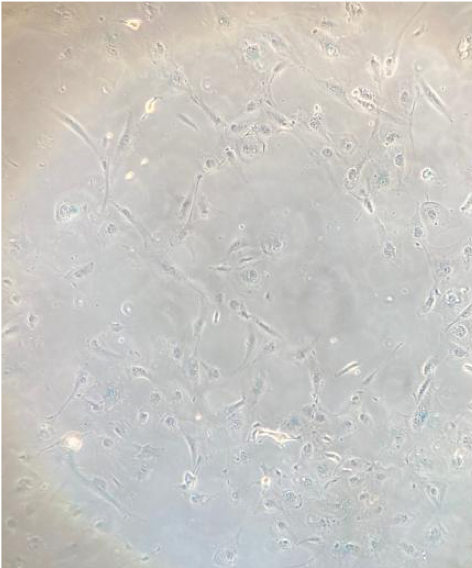

Supplement: Supplementary file 6 — Source data Fig. 5 [file 44321_2024_77_MOESM6_ESM.zip › EMM-2024-19519_SourceDataFor_Figure5/Fugure 5D_Image Data/shPML.png]

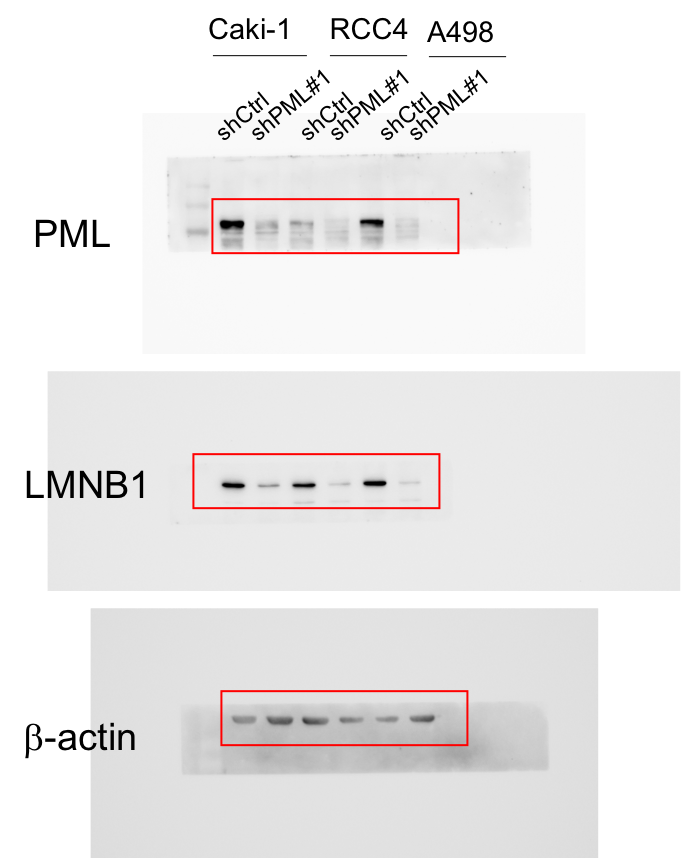

Supplement: Supplementary file 6 — Source data Fig. 5 [file 44321_2024_77_MOESM6_ESM.zip › EMM-2024-19519_SourceDataFor_Figure5/Figure 5B.png]

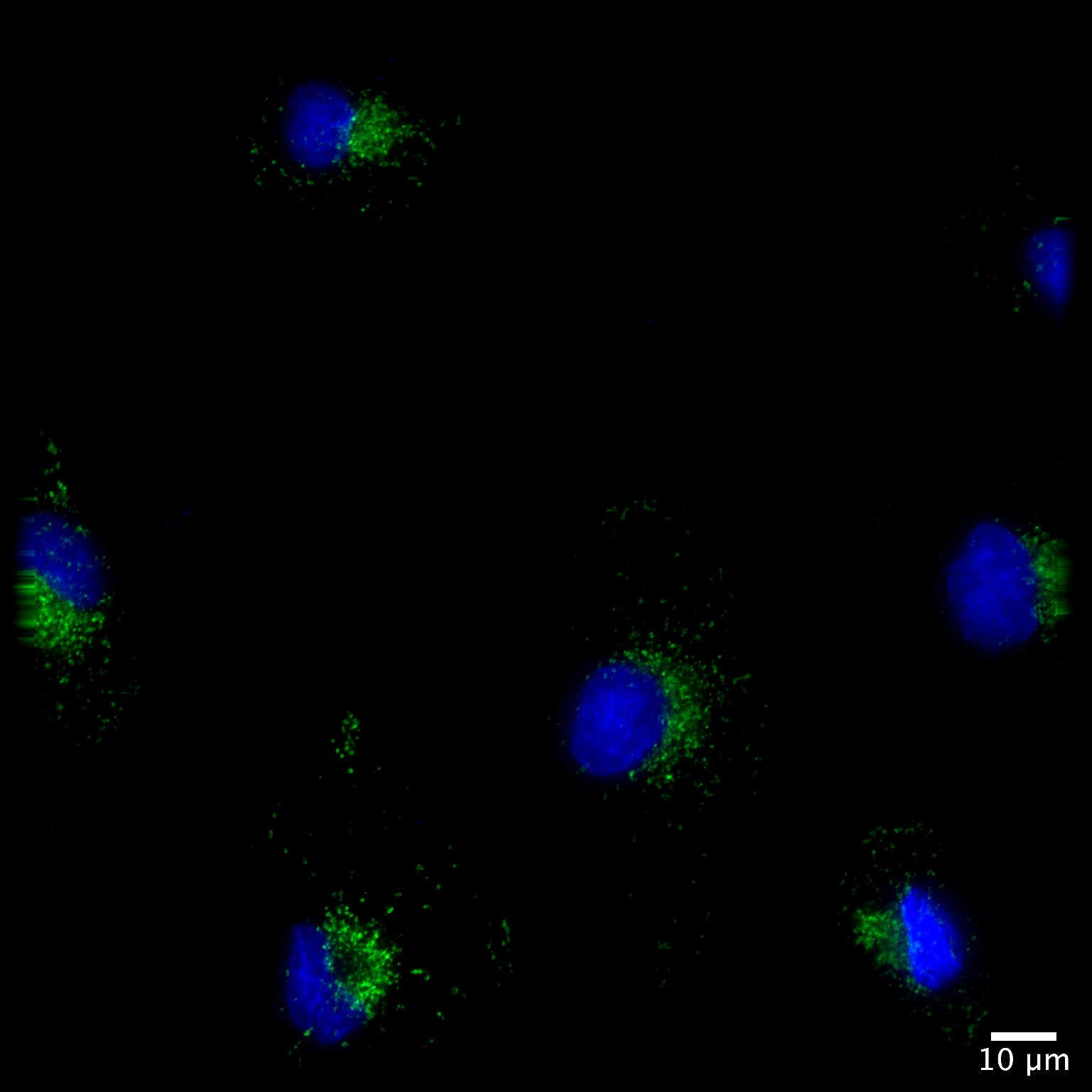

Supplement: Supplementary file 6 — Source data Fig. 5 [file 44321_2024_77_MOESM6_ESM.zip › EMM-2024-19519_SourceDataFor_Figure5/Figure 5C_Image Data/MAX_A498_shCtrl_LAMP2_3_D3D.jpg]

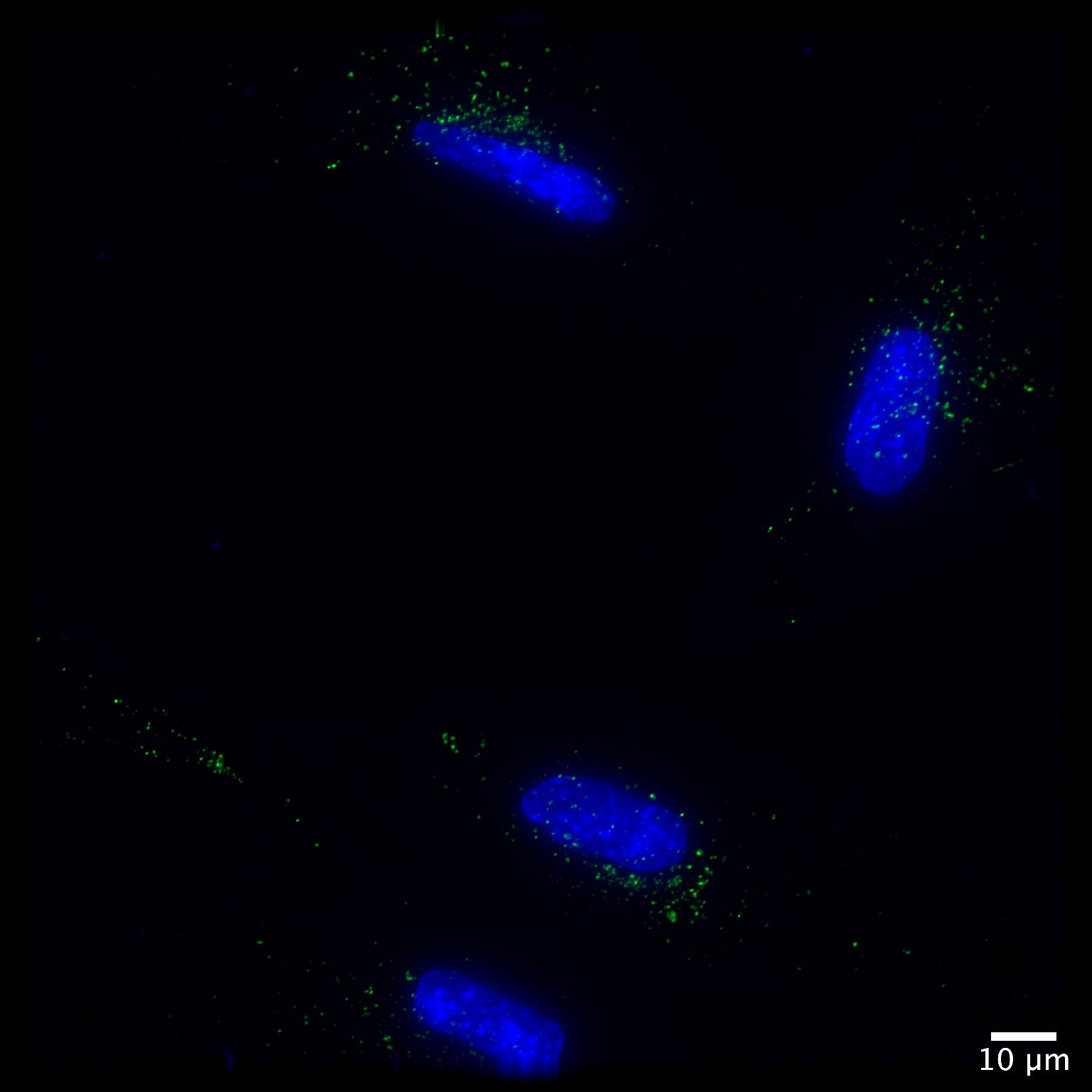

Supplement: Supplementary file 6 — Source data Fig. 5 [file 44321_2024_77_MOESM6_ESM.zip › EMM-2024-19519_SourceDataFor_Figure5/Figure 5C_Image Data/MAX_RCC4_shCtrl_LAMP2_7_D3D.jpg]

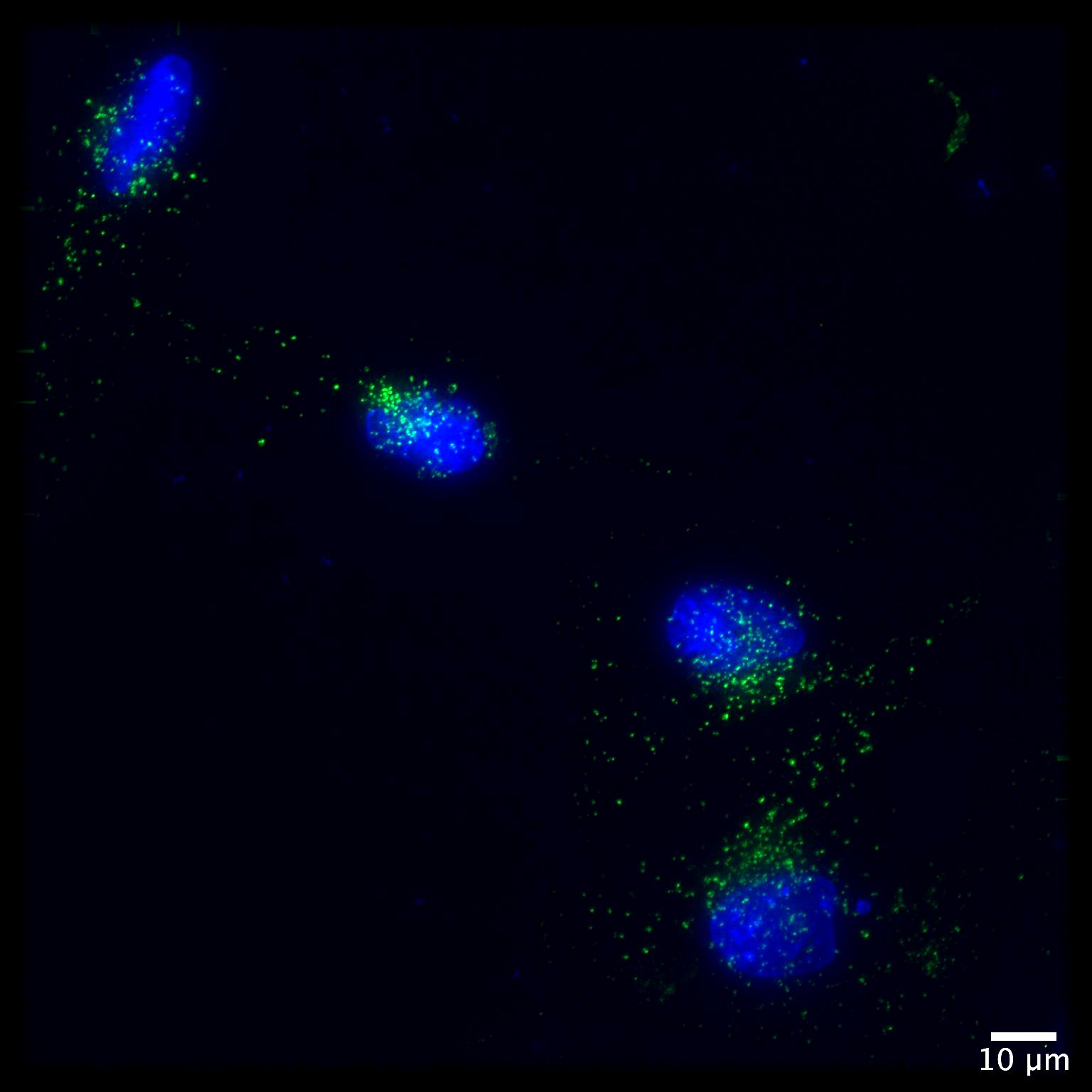

Supplement: Supplementary file 6 — Source data Fig. 5 [file 44321_2024_77_MOESM6_ESM.zip › EMM-2024-19519_SourceDataFor_Figure5/Figure 5C_Image Data/MAX_RCC4_shPML#1_LAMP2_2_D3D.jpg]

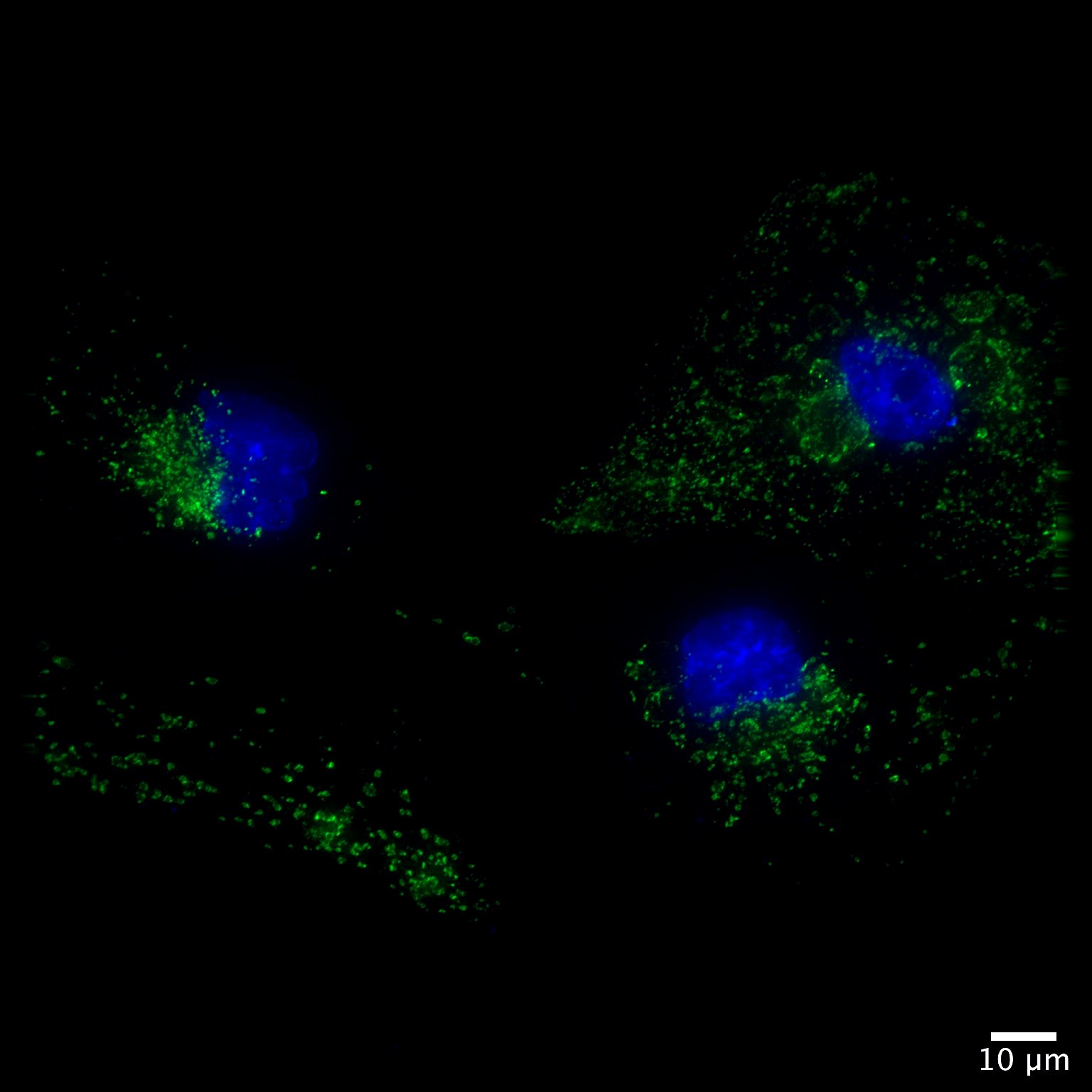

Supplement: Supplementary file 6 — Source data Fig. 5 [file 44321_2024_77_MOESM6_ESM.zip › EMM-2024-19519_SourceDataFor_Figure5/Figure 5C_Image Data/MAX_A498_shPML#1_LAMP2_5_D3D.jpg]

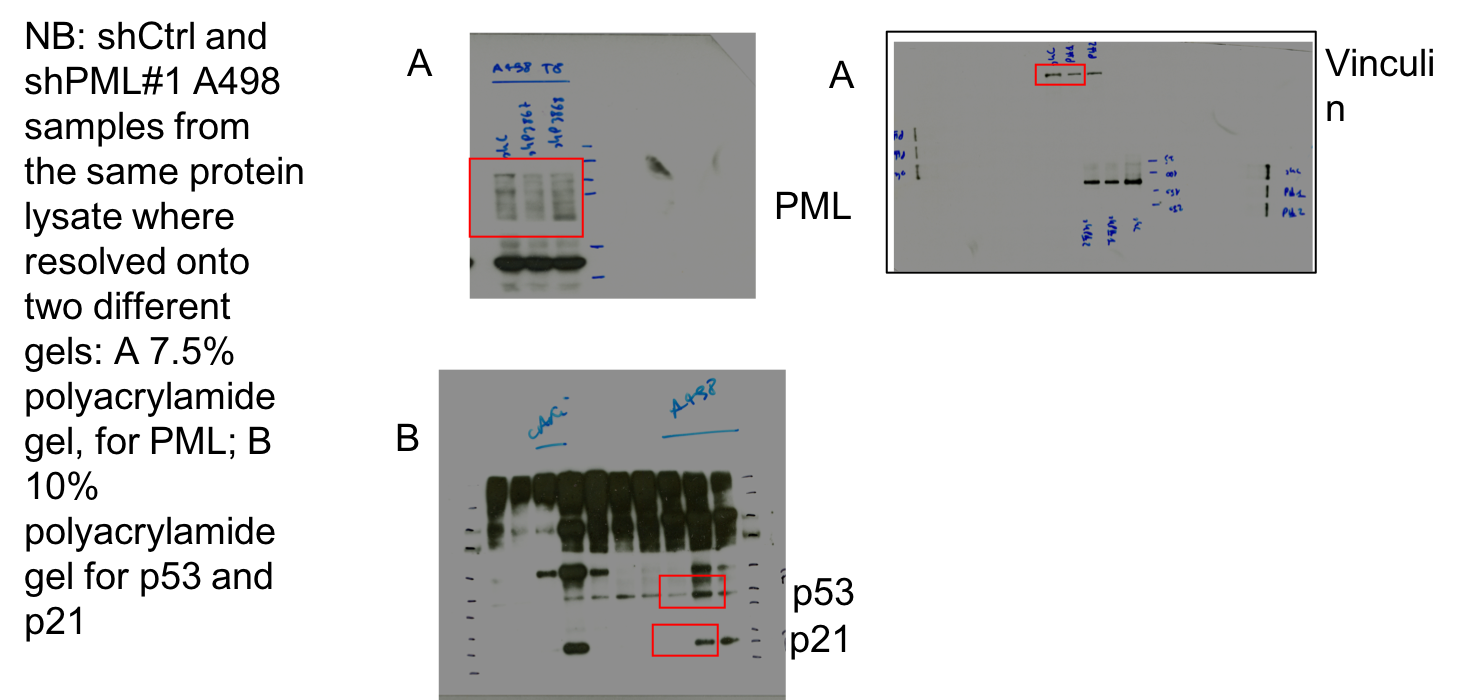

Supplement: Supplementary file 7 — Source data Fig. 6 [file 44321_2024_77_MOESM7_ESM.zip › EMM-2024-19519_SourceDataFor_Figure6/Figure 6A_A498 WB.png]

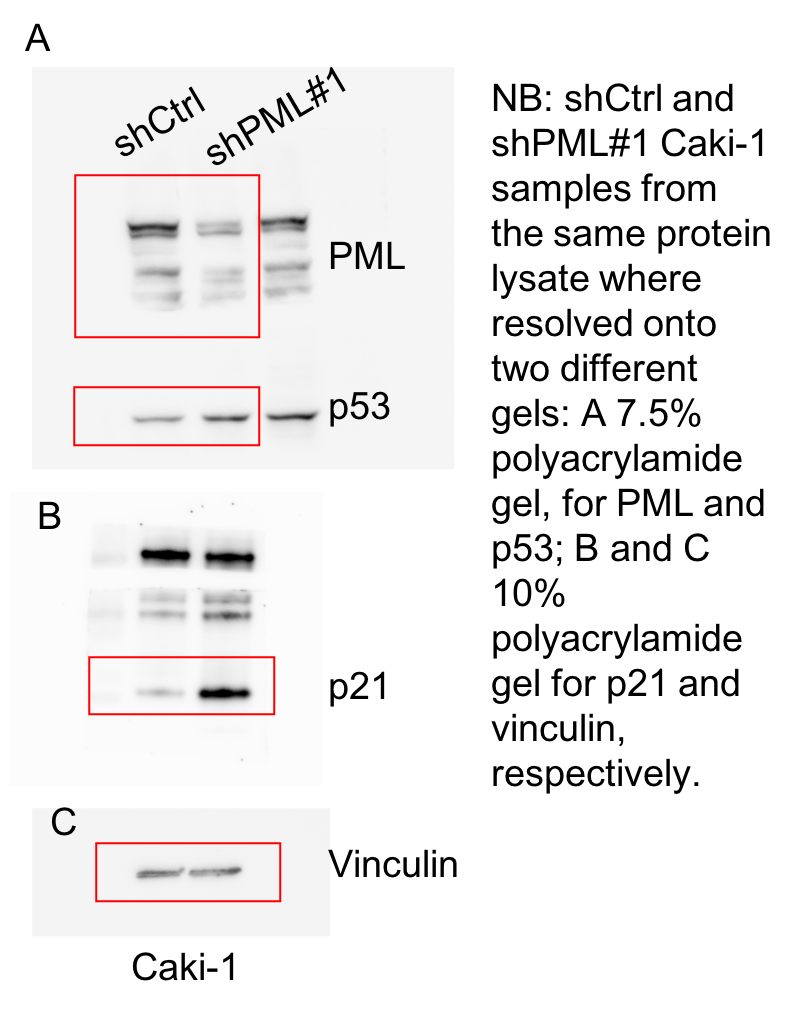

Supplement: Supplementary file 7 — Source data Fig. 6 [file 44321_2024_77_MOESM7_ESM.zip › EMM-2024-19519_SourceDataFor_Figure6/Figure 6A_Caki-1 WB.png]

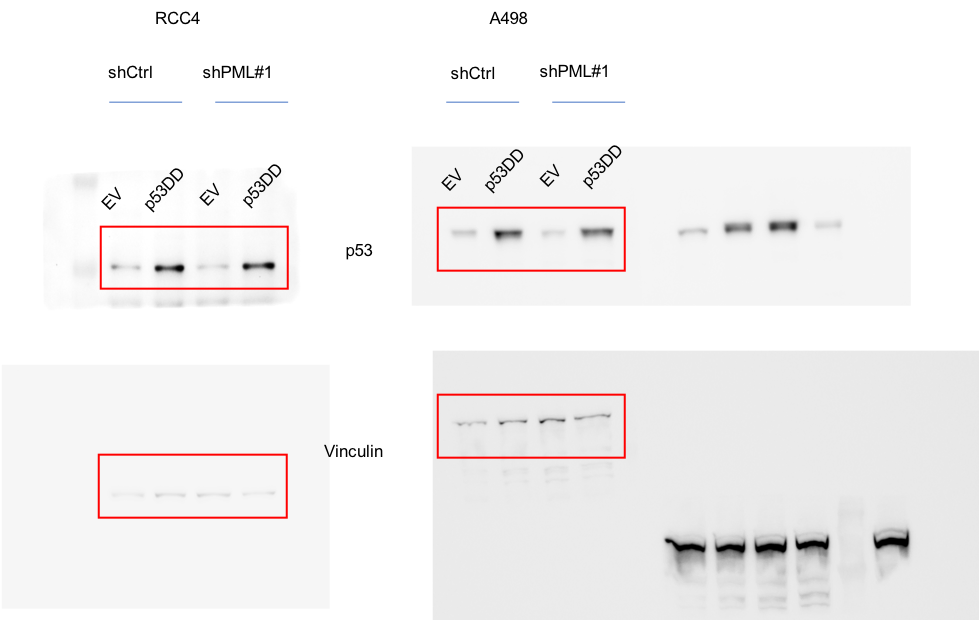

Supplement: Supplementary file 7 — Source data Fig. 6 [file 44321_2024_77_MOESM7_ESM.zip › EMM-2024-19519_SourceDataFor_Figure6/Figure 6B.png]

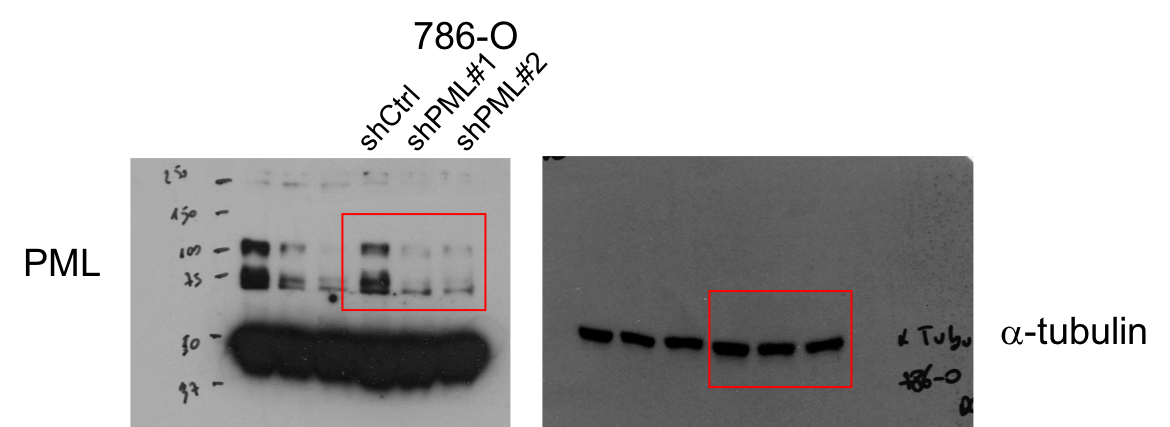

Supplement: Supplementary file 7 — Source data Fig. 6 [file 44321_2024_77_MOESM7_ESM.zip › EMM-2024-19519_SourceDataFor_Figure6/Figure 6D.png]

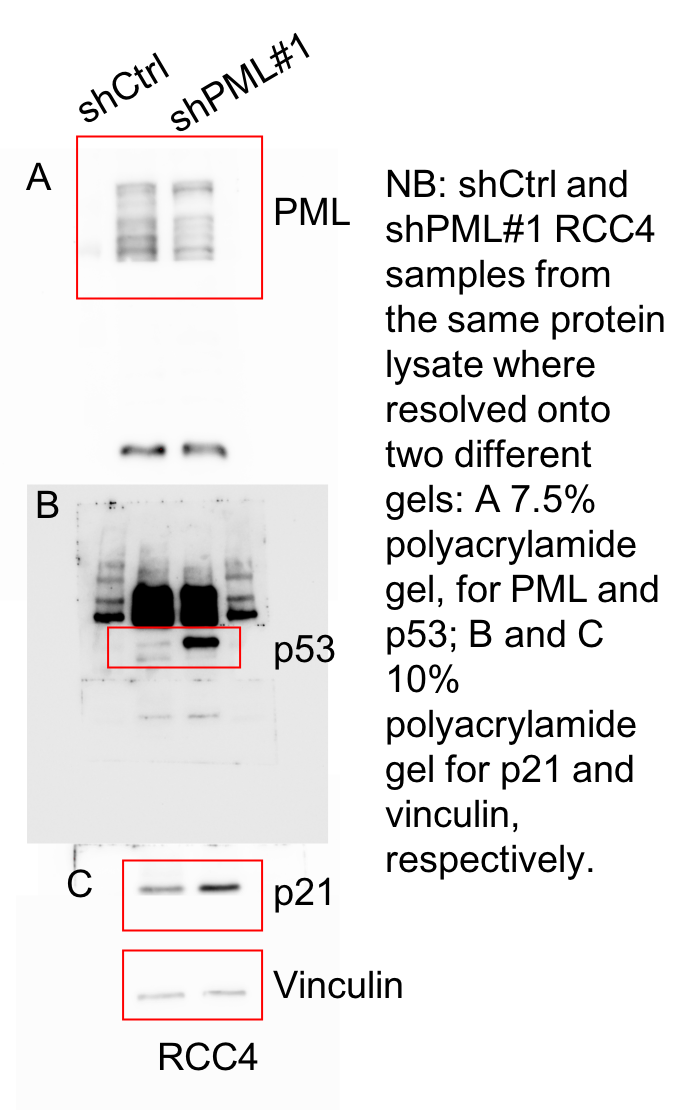

Supplement: Supplementary file 7 — Source data Fig. 6 [file 44321_2024_77_MOESM7_ESM.zip › EMM-2024-19519_SourceDataFor_Figure6/Figure 6A_RCC4 WB.png]

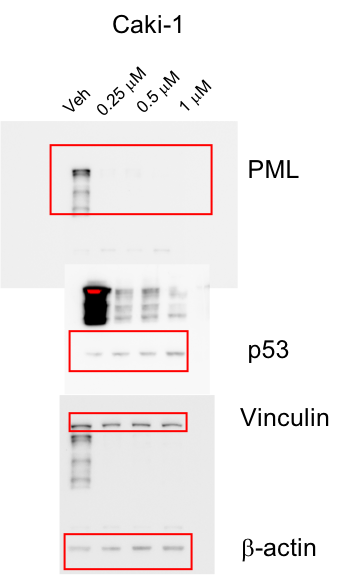

Supplement: Supplementary file 8 — Source data Fig. 7 [file 44321_2024_77_MOESM8_ESM.zip › EMM-2024-19519_SourceDataFor_Figure7/Figure 7A and F.png]

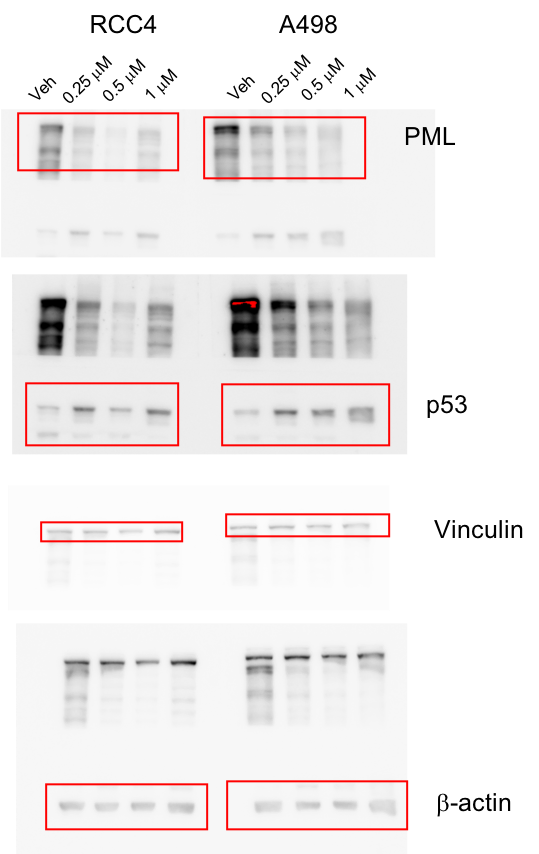

Supplement: Supplementary file 8 — Source data Fig. 7 [file 44321_2024_77_MOESM8_ESM.zip › EMM-2024-19519_SourceDataFor_Figure7/Figure 7B, C and F.png]

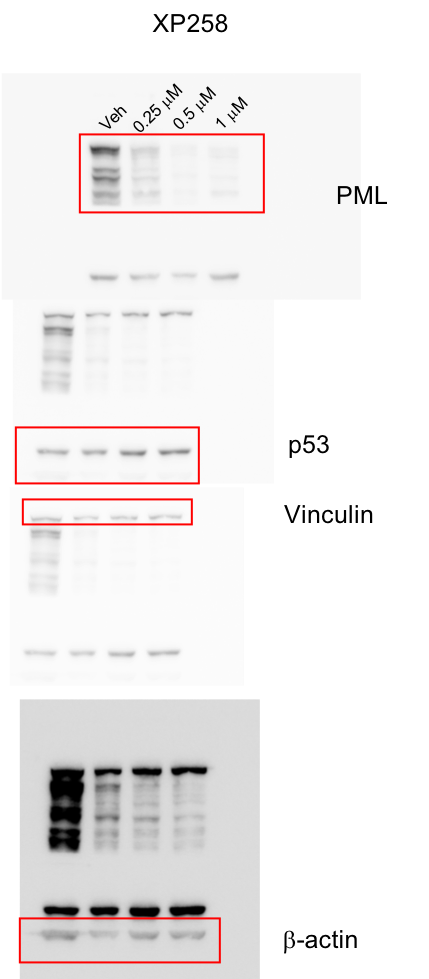

Supplement: Supplementary file 8 — Source data Fig. 7 [file 44321_2024_77_MOESM8_ESM.zip › EMM-2024-19519_SourceDataFor_Figure7/Figure 7D.png]
