# Supplementary material for: PML restrains p53 activity and cellular senescence in clear cell renal cell carcinoma
Source: EMBO Mol Med. 2024 May 10;16(6):7. doi: 10.1038/s44321-024-00077-3 (PMC11178789; doi:10.1038/s44321-024-00077-3)
Supplement: Supplementary file 10 — Expanded View Figures [file 44321_2024_77_MOESM10_ESM.pdf]

Expanded View Figures

Figure EV1. High *PML* mRNA expression correlates with decreased ccRCC survival.

(**A–D**) Kaplan–Meier curves showing the survival probability of patients with high or low PML mRNA in the indicated TCGA datasets: KIRC ($n = 72$ normal and $n = 533$ tumor samples); KIRP ($n = 32$ normal and $n = 290$ tumor samples) GBM ($n = 5$ normal and $n = 156$ tumor samples); HNSC ($n = 44$ normal and $n = 520$ tumor samples). *P* values were obtained by log rank test. (Source: UALCAN). (**E**) Representative images of PML immunofluorescence (green) in the indicated ccRCC and TNBC cell lines. Scale bar 10 µm. Nuclei were counterstained with DAPI (blue). Quantification data showing the distribution of PML-NBs/cell in each cell line ($n = 30$). (**F–I**) Box and whisker plots with individual data points showing the distribution of nuclear area (**E**), PML nuclear intensity (**F**), number of PML-NBs/nucleus (**G**) and number of PML-NBs per unit area (**H**) in RCC4 and MDA-MB-231 cells (RCC4, $n = 124$; MDA-MB-231, $n = 150$). The central band denotes the median value, box contains interquartile ranges, while whiskers mark minimum and maximum values. Statistical significance was calculated with Bonferroni corrected Kolmogorov–Smirnov test.

© The Author(s)

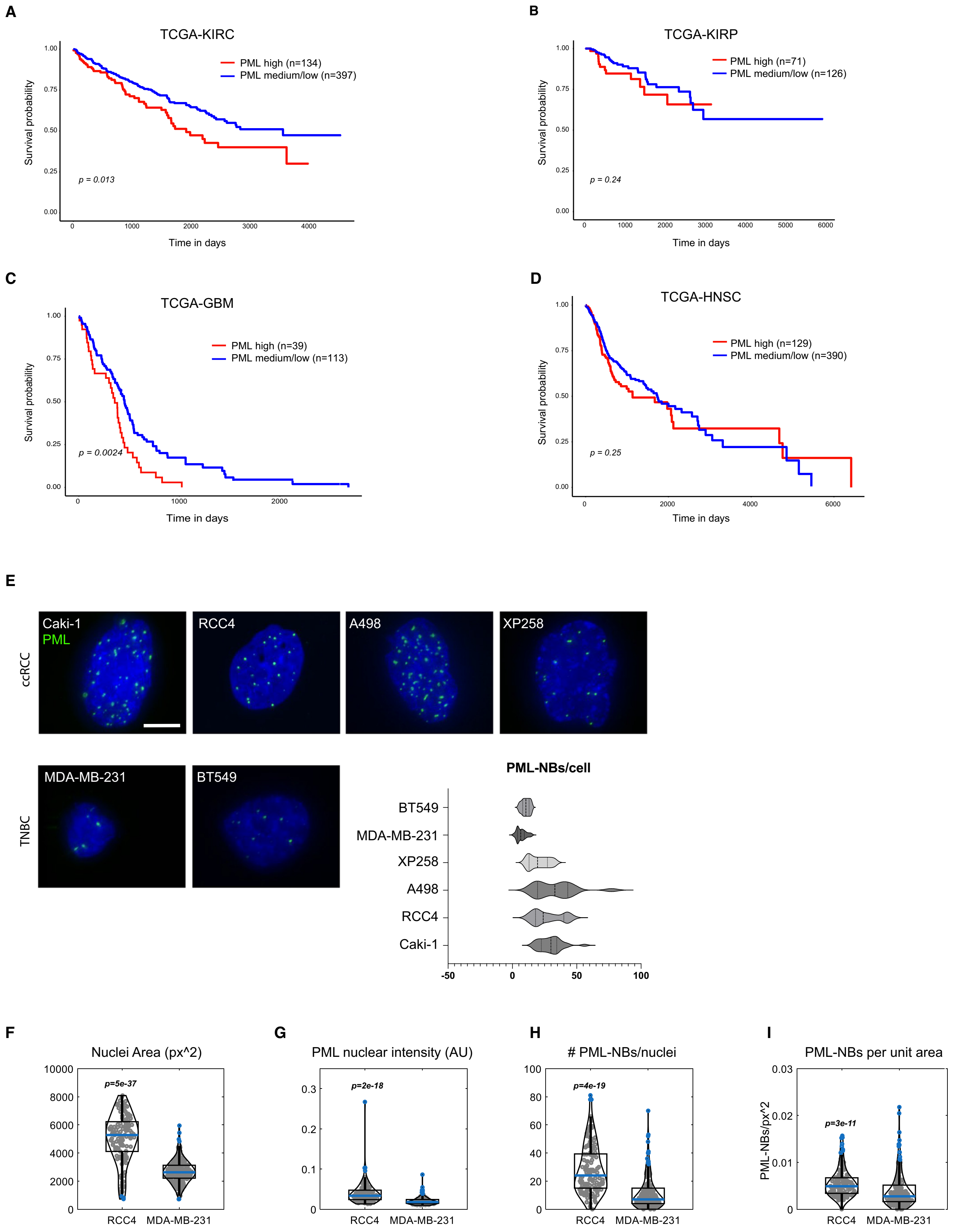
A
TCGA-KIRC
PML high (n=134)
PML medium/low (n=397)
Survival probability
p = 0.013
Time in days
B
TCGA-KIRP
PML high (n=71)
PML medium/low (n=126)
Survival probability
p = 0.24
Time in days
C
TCGA-GBM
PML high (n=39)
PML medium/low (n=113)
Survival probability
p = 0.0024
Time in days
D
TCGA-HNSC
PML high (n=129)
PML medium/low (n=390)
Survival probability
p = 0.25
Time in days
E
ccRCC
Caki-1
PML
RCC4
A498
XP258
TNBC
MDA-MB-231
BT549
PML-NBs/cell
BT549
MDA-MB-231
XP258
A498
RCC4
Caki-1
F
Nuclei Area (px^2)
p=5e-37
RCC4
MDA-MB-231
G
PML nuclear intensity (AU)
p=2e-18
RCC4
MDA-MB-231
H
# PML-NBs/nuclei
p=4e-19
RCC4
MDA-MB-231
I
PML-NBs per unit area
PML-NBs/px^2
p=3e-11
RCC4
MDA-MB-231

© The Author(s)

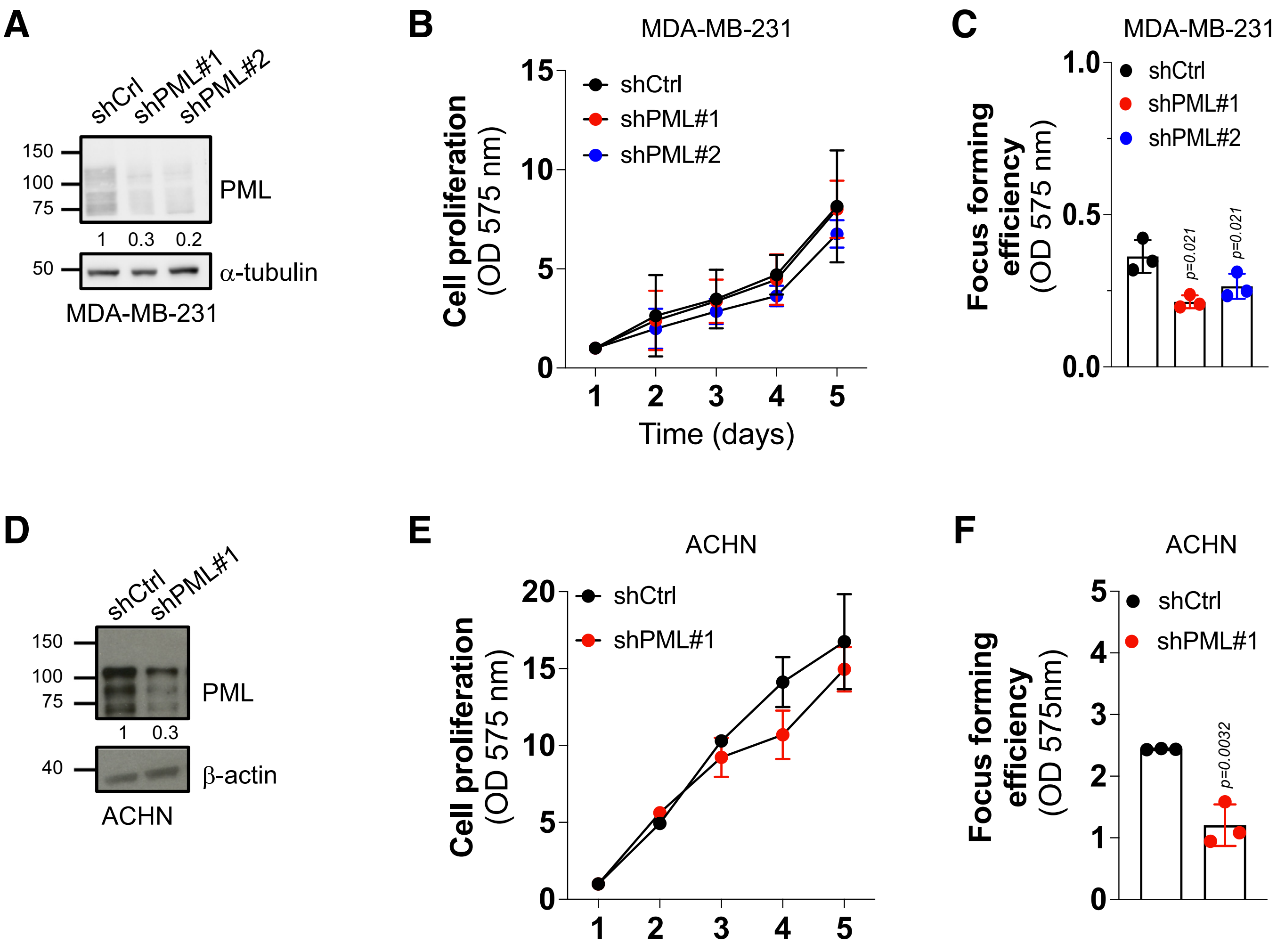

Figure EV2. PML silencing in TNBC and papillary RCC cell lines affects focus forming efficiency.

(**A**) Immunoblot analysis showing silencing efficiency of two independent shRNAs against PML (shPML#1 and shPML#2) compared to a scrambled shRNA sequence (shCtrl) in MDA-MB-231 cells. β-actin was used as loading control. Numbers represent densitometric analysis of PML levels normalized over α-tubulin. Molecular weight markers (kDa) are shown on the left. The blot represents one out of three independent experiments with similar results. (**B**, **C**) Proliferation (**B**) and focus-forming (**C**) assays performed in MDA-MB-231 cell line expressing shPML#1, shPML#2, or shCtrl. Data represent mean ± SD of three biological replicates (Student's *t* test).
(**D**) Immunoblot analysis showing silencing efficiency of shPML#1 compared to shCtrl in ACHN cell line. β-actin was used as loading control. Numbers represent densitometric analysis of PML levels normalized over α-tubulin. Molecular weight markers (kDa) are shown on the left. The blot represents one out of three independent experiments with similar results. (**E**, **F**) Proliferation (**E**) and focus-forming (**F**) assays performed in ACHN cell line expressing shPML#1 or shCtrl. Data represent mean ± SD of three biological replicates (Student's *t* test).

© The Author(s)

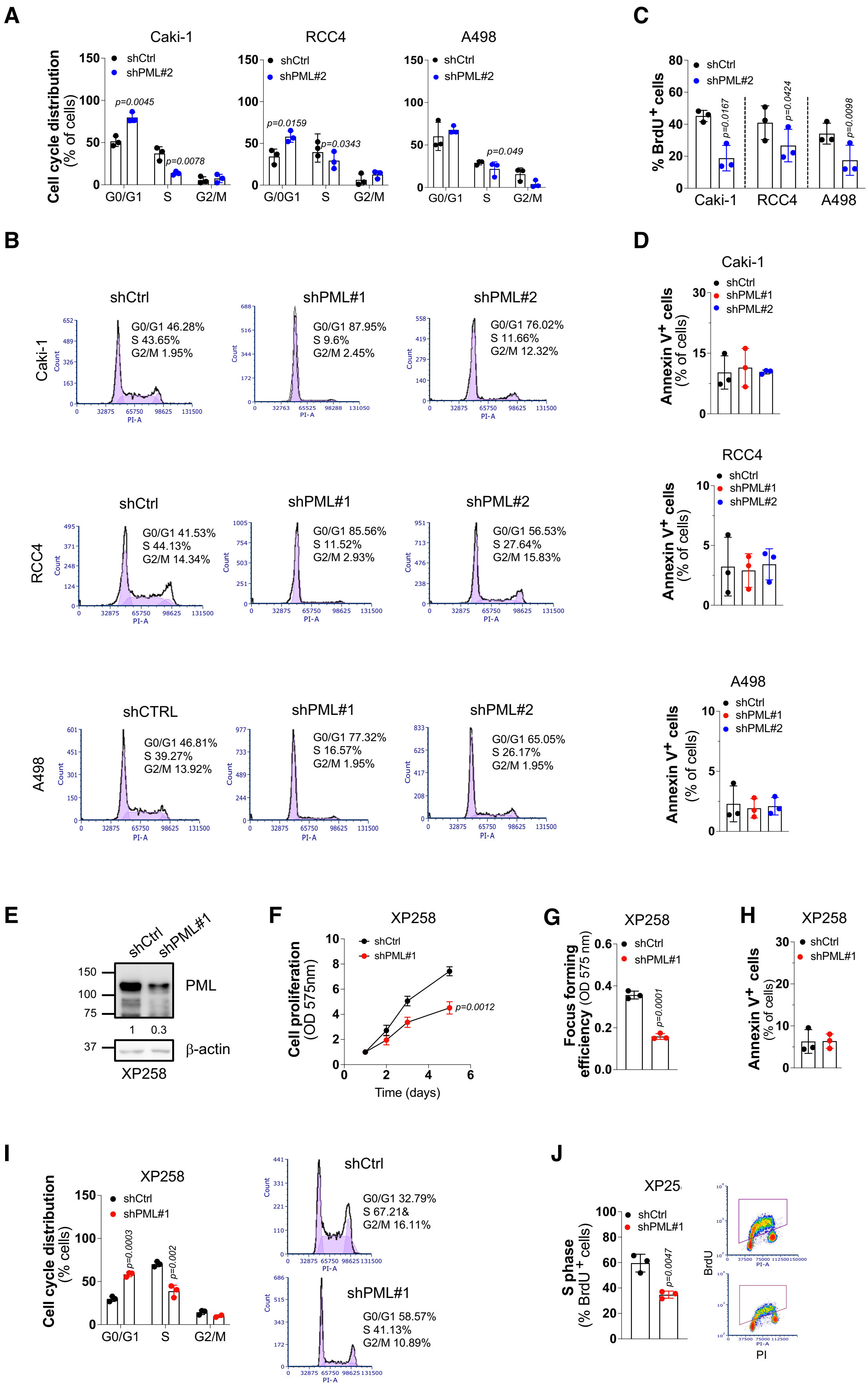
A
Caki-1
RCC4
A498
Cell cycle distribution (% of cells)
shCtrl
shPML#2
p=0.0045
p=0.0078
p=0.0159
p=0.0343
p=0.049
G0/G1
S
G2/M
G/0G1
C
% BrdU+ cells
p=0.0167
p=0.0424
p=0.0098
Caki-1
RCC4
A498
B
shCtrl
shPML#1
shPML#2
Caki-1
G0/G1 46.28%
S 43.65%
G2/M 1.95%
G0/G1 87.95%
S 9.6%
G2/M 2.45%
G0/G1 76.02%
S 11.66%
G2/M 12.32%
RCC4
G0/G1 41.53%
S 44.13%
G2/M 14.34%
G0/G1 85.56%
S 11.52%
G2/M 2.93%
G0/G1 56.53%
S 27.64%
G2/M 15.83%
A498
shCTRL
G0/G1 46.81%
S 39.27%
G2/M 13.92%
G0/G1 77.32%
S 16.57%
G2/M 1.95%
G0/G1 65.05%
S 26.17%
G2/M 1.95%
Count
PI-A
D
Annexin V+ cells (% of cells)
E
shCtrl
shPML#1
PML
1 0.3
β-actin
XP258
F
XP258
Cell proliferation (OD 575nm)
p=0.0012
Time (days)
G
Focus forming efficiency (OD 575 nm)
p=0.0001
H
Annexin V+ cells (% of cells)
I
XP258
Cell cycle distribution (% cells)
p=0.0003
p=0.002
G0/G1 32.79%
S 67.21&
G2/M 16.11%
G0/G1 58.57%
S 41.13%
G2/M 10.89%
J
S phase (% BrdU+ cells)
p=0.0047
BrdU
PI

 © The Author(s)

Figure EV3. PML silencing inhibits ccRCC proliferation and cell cycle progression.

(**A**) Cell cycle distribution by FACS in the indicated ccRCC cell lines expressing shPML#2, or shCtrl. Data represent mean ± SD of three biological replicates (Student's *t* test). (**B**) Representative FACS profiles of the indicated cell lines expressing shPML#1, shPML#2 or shCtrl. Shown are the results of one out of three independent experiments with similar results. (**C**) Percentage of BrdU-positive cells in the indicated ccRCC cell lines expressing shPML#2 or shCtrl. Data represent mean ± SD of three biological replicates (Student's *t* test). (**D**) Percentage of Annexin-V positive cells in the indicated cell lines expressing shPML#1, shPML#2 or shCtrl. Data represent mean ± SD of three biological replicates (Student's *t* test). (**E**) Immunoblot analysis showing silencing efficiency of shPML#1 in XP258 PDX-derived cell line. β-actin was used as loading control. Numbers represent densitometric analysis of PML levels normalized over α-tubulin. Molecular weight markers (kDa) are shown on the left. The blot represents one out of three independent experiments with similar results. (**F**, **G**) Proliferation (**F**) and focus-forming (**G**) assays performed in XP258 PDX-derived cell line with shPML#1 or shCtrl. Data represent mean ± SD of three biological replicates (Student's *t* test). (**H**) Percentage of Annexin-V positive cells in XP250 PDX-derived cell line expressing shPML#1 or shCtrl. Data represent mean ± SD of three biological replicates (Student's *t* test). (**I**) Cell cycle distribution by FACS of XP258 PDX-derived cell line expressing shPML#1 or shCtrl (left). Data represent mean ± SD of three biological replicates (Student's *t* test). Representative FACS profiles of cell cycle distribution of XP258 PDX-derived cell line expressing shPML#1 or shCtrl (right). Shown are the results of one out of three independent experiments with similar results. (**J**) Percentage of BrdU-positive cells in XP258 cell line expressing shCtrl or shPML#1 (left). Representative scatter plot of BrdU postitive cells by FACS analysis (right). Shown are the results of one out of three independent experiments with similar results. In the left panel, data represent mean ± SD of three biological replicates (Student's *t* test).

© The Author(s)

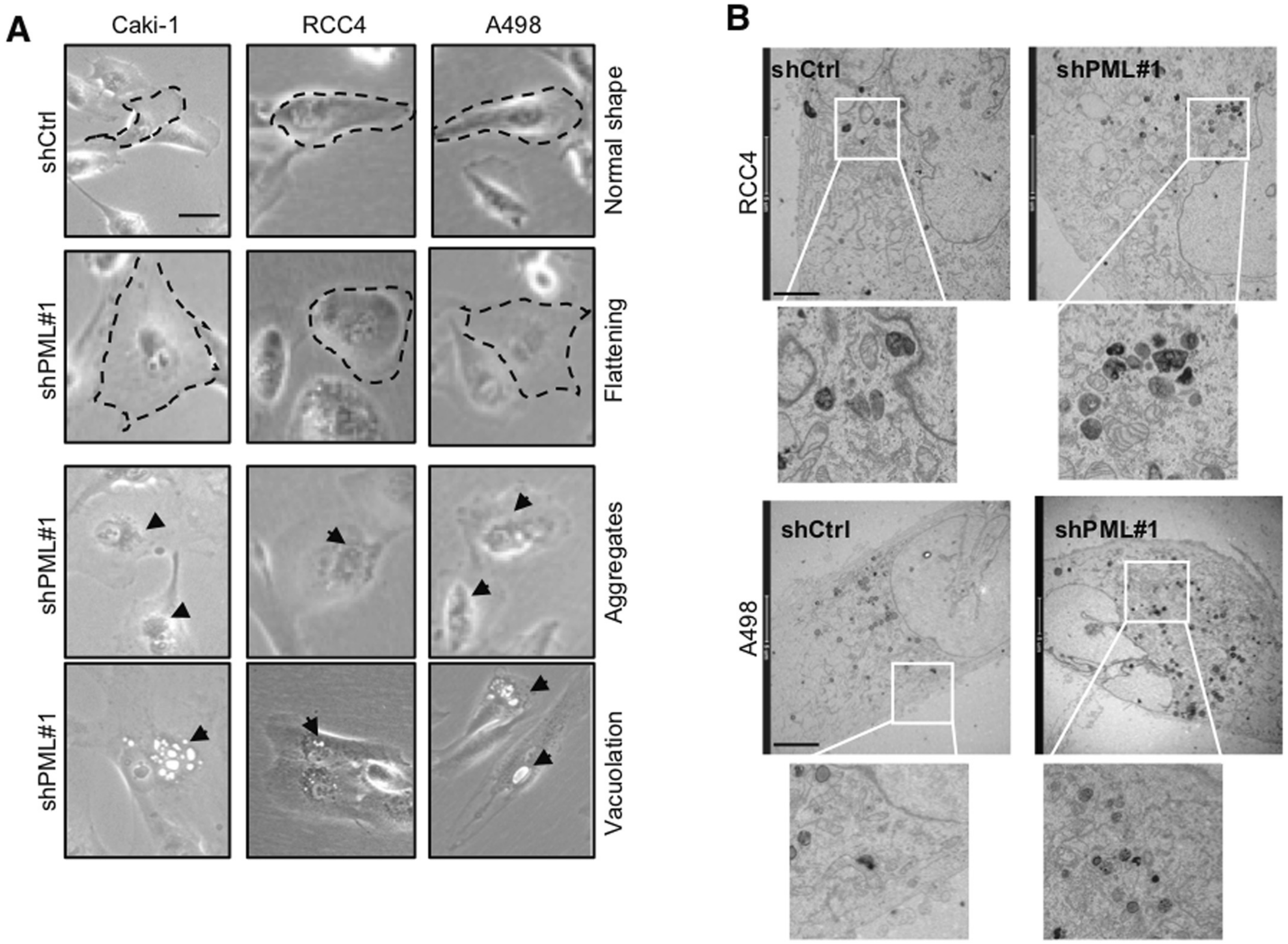

Figure EV4. PML knockdown recapitulates morphological features of cellular senescence.

(**A**) Representative phase contrast images of the indicated cell lines expressing shPML#1 or shCtrl. Black arrowheads indicate cytoplasmic aggregates and vacuoles. Scale bar 20 µm. Shown are the results of one out of three experiments with similar results. (**B**) Representative transmission electron microscopy of RCC4 and A498 cell lines expressing shPML#1 or shCtrl. Indents show higher magnifications of degradative structures in boxed areas. Scale bar 5 µm. Shown are the results of one out of two independent experiments with similar results.

 © The Author(s)

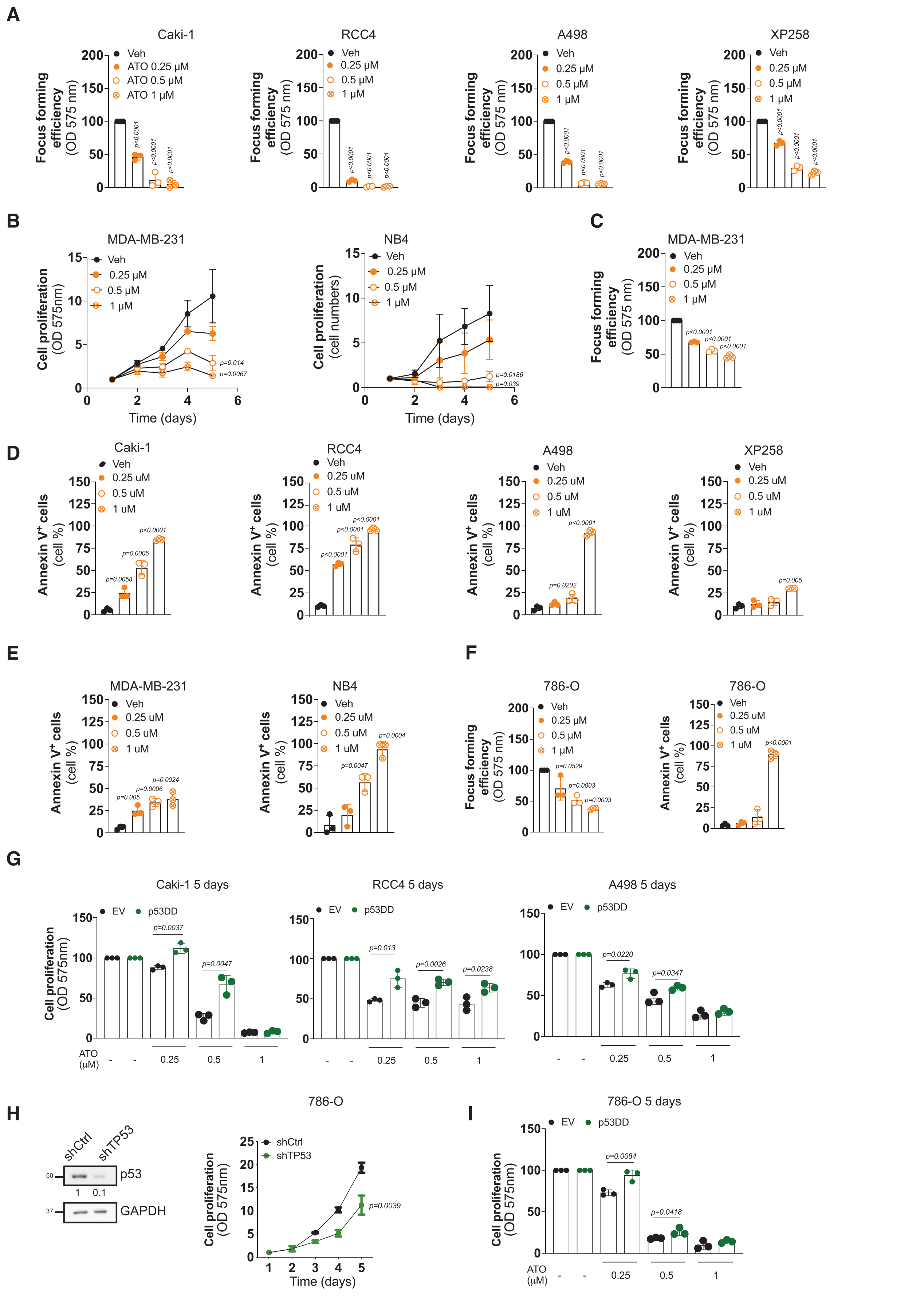
A
Caki-1
RCC4
A498
XP258
Focus forming efficiency (OD 575 nm)
Veh
ATO 0.25 μM
ATO 0.5 μM
ATO 1 μM
0.25 μM
0.5 μM
1 μM
B
MDA-MB-231
NB4
Cell proliferation (OD 575nm)
Cell proliferation (cell numbers)
Time (days)
C
MDA-MB-231
D
Caki-1
RCC4
A498
XP258
Annexin V+ cells (cell %)
Veh
0.25 uM
0.5 uM
1 uM
E
MDA-MB-231
NB4
F
786-O
786-O
G
Caki-1 5 days
RCC4 5 days
A498 5 days
EV
p53DD
ATO (μM)
H
shCtrl
shTP53
p53
GAPDH
786-O
I
786-O 5 days

Figure EV5. ATO inhibits cell proliferation and induces apoptosis in ccRCC, TNBC and APL cells.

(**A**) Focus-forming efficiency of the indicated cell lines upon 14 days of ATO treatment (0.25–1 µM). Data represent mean ± SD of three independent experiments (Student's *t* test). (**B**, **C**) Cell proliferation of MDA-MB-231 and NB4 cells (**B**) and focus-forming assay of MDA-MB-231 cells (**C**) upon 5 days (**B**) and 14 days (**C**) of ATO treatment (0.25–1 µM). For MDA-MB-231 cells, data are shown as fold change of OD 575 nm measurements over day 1. For NB4 cells, data are shown as normalized cell numbers over day 1. Data represent mean ± SD of three independent experiments (Student's *t* test). (**D**, **E**) Percentage of Annexin-V positive cells of the indicated cell lines upon 5 days of ATO treatment (0.25–1 µM). Data represent mean ± SD of three independent experiments (Student's *t* test). (**F**) Focus-forming assay of 786-O cells upon 14 days of ATO (0.25–1 µM) treatment is shown on the left. Percentage of 786-O Annexin-V positive cells upon 5 days of ATO (0.25–1 µM) treatment is shown on the right. Data represent mean ± SD of three independent experiments (Student's *t* test). (**G**) Cell proliferation of the indicated cell lines upon 5 days of treatment with ATO (0.25–1 µM). Data are represented as normalized values of OD 575 nm measurements over vechicle-treated cells. Data represent mean ± SD of three independent experiments (Student's *t* test). (**H**) Immunoblot analysis showing the silencing efficiency of TP53 shRNA (shTP53) compared to a scramble sequence (shCtrl) in 786-O cells (left). GAPDH was used as loading control. Numbers represent densitometric analysis of p53 levels normalized over GAPDH. The blot represents one out of three independent experiments with similar results. Proliferation assay of 786-O cells expressing shCtrl or shTP53 (right). Data are shown as fold change of OD 575 nm measurements over day 1. Data represent mean ± SD of three independent experiments (Student's *t* test). (**I**) Cell proliferation of 786-O cells upon 5 days of treatment with ATO (0.25–1 µM). Data are represented as normalized values of OD 575 nm measurements over vechicle-treated cells. Data represent mean ± SD of three independent experiments (Student's *t* test).

© The Author(s)
